# Characterization of Methane Excess and Absolute Adsorption in Various Clay Nanopores from Molecular Simulation

**DOI:** 10.1038/s41598-017-12123-x

**Published:** 2017-09-20

**Authors:** Yuanyuan Tian, Changhui Yan, Zhehui Jin

**Affiliations:** 1 State Key Laboratory of Oil and Gas Reservoir Geology and Exploitation (Chengdu University of Technology), Chengdu, 610059 Sichuan P.R. China; 2 College of Energy, Chengdu University of Technology, Chengdu, 610059 Sichuan P.R. China; 3 School of Mining and Petroleum Engineering, Faculty of Engineering, University of Alberta, Edmonton, T6G 1H9 Canada

## Abstract

In this work, we use grand canonical Monte Carlo (GCMC) simulation to study methane adsorption in various clay nanopores and analyze different approaches to characterize the absolute adsorption. As an important constituent of shale, clay minerals can have significant amount of nanopores, which greatly contribute to the gas-in-place in shale. In previous works, absolute adsorption is often calculated from the excess adsorption and bulk liquid phase density of absorbate. We find that methane adsorbed phase density keeps increasing with pressure up to 80 MPa. Even with updated adsorbed phase density from GCMC, there is a significant error in absolute adsorption calculation. Thus, we propose to use the excess adsorption and adsorbed phase volume to calculate absolute adsorption and reduce the discrepancy to less than 3% at high pressure conditions. We also find that the supercritical Dubinin-Radushkevich (SDR) fitting method which is commonly used in experiments to convert the excess adsorption to absolute adsorption may not have a solid physical foundation for methane adsorption. The methane excess and absolute adsorptions per specific surface area are similar for different clay minerals in line with previous experimental data. In mesopores, the excess and absolute adsorptions per specific surface area become insensitive to pore size. Our work should provide important fundamental understandings and insights into accurate estimation of gas-in-place in shale reservoirs.

## Introduction

Coupling with the growing global energy demands and continuous depletion of conventional energy resources, as an important unconventional energy supply, shale gas has garnered more and more attentions in recent years^1–4^. Comparing to conventional reservoirs, shale has a heterogeneous composition consisting of organic and inorganic matters. Organic matter is mainly composed of kerogen, while inorganic materials consist of clay minerals, quartz, and calcite, etc. Due to large amount of nanoscale pores, adsorbed gas can provide a significant source for gas-in-place in shale. It is reported that the adsorbed gas may reach 85% of gas-in-place in shale reservoir^3^. Both kerogen and clay minerals can have significant amount of nanopores^5^. According to shale component analysis, the amount of clay minerals can be in the range from 25% to 70%^6^. Recently, it is reported that clay minerals provide significant amount of nanopores and specific surface area for the transitional shales in China^7^. Experimental researches have shown that clay minerals can significantly contribute to adsorbed gas capacity^8–10^.

There have been a number of experimental studies on gas adsorption in clay minerals and its contribution to gas-in-place in shale. Ross and Bustin compared adsorption capacities of various clay minerals on both dry and moisture basis^11^. They reported that illite and montmorillonite have larger adsorption capacities than kaolinite at dry condition, but opposite is true for moisturized condition. Liu and his coworkers found that montmorillonite has larger adsorption capacity than kaolinite, while illite has the smallest adsorption capacity^12^, which is in line with Zhang *et al*.^13^ and Ji *et al*.^14^. In addition, Ji *et al*.^14^ found that the specific surface area (SSA) is the main control on methane adsorption capacity in various clay minerals.

**Figure 1:**
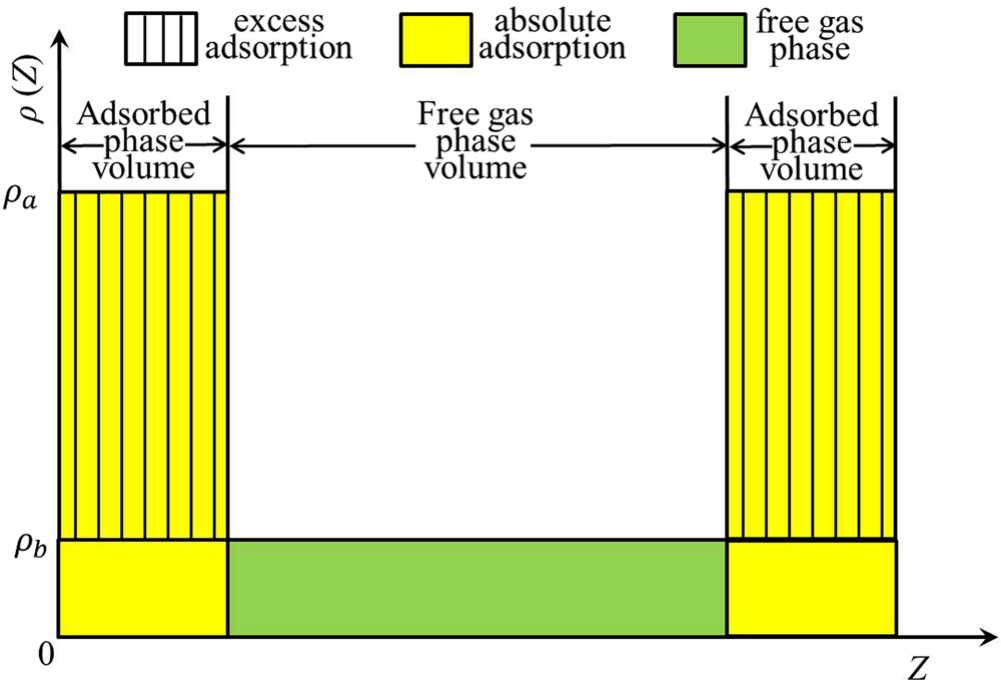
The schematic representation of free gas, excess adsorption, and absolute adsorption of methane in slit-nanopores. The yellow area represents adsorbed phase and the green area depicts free gas phase, respectively.

In experiment, there are two commonly used methods to study gas adsorption: gravimetric and volumetric method. Gravimetric method uses magnetic suspension balance to obtain adsorption isotherms^15^. It measures the excess adsorption capacity ${m}_{ex}$ based on the difference between gravity and buoyancy^16^. In practice, it is difficult to assess the buoyancy accurately. As a result, gravimetric method may become unsuitable to obtain the total gas uptake ${m}_{tot}$. On the other hand, volumetric method can measure ${m}_{tot}$ in porous media^17^ and ${m}_{ex}$ is obtained by subtracting the amount of free gas in total accessible pore volume ${V}_{p}$ from ${m}_{tot}$,^18,19^:

1 $${m}_{ex}={m}_{tot}-{\rho }_{b}{V}_{p},$$

where ${\rho }_{b}$ is bulk gas density. Methane adsorption in nanopores is generally considered as single-layered adsorption as we will show in section 3.2^20^. When pore size is large, methane density profile in the middle of pores approaches bulk density^21^. As a result, one can assume that methane adsorption in nanopore can be divided into adsorbed and free gas regions as shown in Fig. 1. While free gas has a density of ${\rho }_{b}$, adsorbed phase density ${\rho }_{a}$ can be higher than ${\rho }_{b}$. Absolute adsorption ${m}_{abs}$ is defined as the adsorbed amount in the adsorbed phase (yellow area in Fig. 1)^22^. Based on the adsorption model shown in Fig. 1, only the adsorbed phase contributes to ${m}_{abs}$ and ${m}_{ex}$. With these assumptions, ${m}_{abs}$ can be obtained from ${m}_{ex}$ and ${\rho }_{a}$ ^4,23,24^,

2 $${m}_{abs}=\frac{{m}_{ex}}{1-{\rho }_{b}/{\rho }_{a}}.$$

The key to calculate ${m}_{abs}$ in Eq. (2) is to accurately obtain ${\rho }_{a}$. In previous works, ${\rho }_{a}$ was assumed to be a constant as the liquid density of methane at normal boiling point, 420 kg/m^3^ ^25–27^. However, it is well known that adsorbed phase density changes with pressure and temperature^21^. Gensterblum *et al*.^28–30^ obtained ${\rho }_{a}$ by using a least-squares minimization procedure based on the modified Langmuir equation and Dubinin-Radushkevitch (DR) equation to match excess adsorption data.

Recently, Xiong *et al*.^31^ used Langmuir, Supercritical DR (SDR), and Ono-Kondo model to correct the excess adsorption to the absolute adsorption assuming constant ${\rho }_{a}$ as the density of liquid methane at boiling temperature, 420 kg/m^3^ or the methane density at the critical point, 373 kg/m^3^. However, these methods are rather a pure curve fitting without reliable physical mechanisms. Another way to obtain ${m}_{abs}$ is based on the adsorbed phase volume ${m}_{ex}$ and ${V}_{a}$ ^4,32^,

3 $${m}_{abs}={m}_{ex}+{\rho }_{b}{V}_{a}.$$

Zhang *et al*.^33^ assumed that absolute adsorption stays constant at high pressures and calculated ${V}_{a}$ by taking derivative of excess adsorption with respect to the bulk density. However, there is no rigorous proof of constant methane absolute adsorption even at high pressure conditions.

Comparing to experimental measurements, molecular simulation can explore wider range of pressure and temperature conditions^34^ and provide underlying mechanisms of gas adsorption from molecular perspective. There have been a number of molecular simulation works on methane adsorption in clay minerals and the calculation of excess and absolute adsorption. Jin and Firoozabadi studied methane adsorption in montmorillonite clay and found that adsorption is mainly dominated by surface area^21^. Chen *et al*.^34^ and Xiong *et al*.^35^ found that excess adsorption capacity of methane in various clay nanopores decreases with pore size. In another work, Chen *et al*.^36^ reported that CO_2_ and N_2_ excess adsorption in mesopores does not change with pore size. On the other hand, Zhang *et al*.^33^ reported that at high pressures, methane excess adsorption can be negative. They also defined the saturated adsorbed phase density ${\rho }_{a,s}$ obtained from the linear intercept of the excess adsorption with the bulk gas density at pressure ${P}_{s}$. They assumed that ${\rho }_{a}$ becomes constant when the pressure is higher than ${P}_{s}$. When pressure is lower than ${P}_{s}$, ${\rho }_{a}$ is obtained from the Langmuir curve fitting to ${\rho }_{a,s}$. However, the excess adsorption is very sensitive to pore volume^37^. Varying excess adsorption can be obtained based on different pore volume characterizations^38^. Thus, the accurate characterization of pore volume is essential for molecular simulation to compare with experimental measurement. In addition, calculation of the adsorbed phase density/volume and absolute adsorption still remains as a daunting challenge for scientists and engineers.

In this work, we use grand canonical Monte Carlo (GCMC) simulations^21,34,36,38^ to study methane adsorption in illite, montmorillonite, and kaolinite nanopores. X-ray diffraction reveals that they are the main constituent of clay minerals in shale^12,39^. These three clay minerals are represented by full atomistic models and methane molecules are depicted by the single-site Lennard Jones (LJ) particles. We explicitly consider intermolecular interactions between methane molecules and clay atoms. We assume that the inter-pore interactions are negligible and methane adsorbs in nanometer slit-like pores^21^, which is one of the main pore shapes in shale reservoirs^40–42^. Based on low pressure nitrogen adsorption^43,44^, in addition to slit-like pores, shale can have other shaped pores such as ink-bottle^45,46^. Although the adsorption behavior and density distributions may be different in various pore geometries, the focus of this work is to characterize the excess and absolute adsorption in a slit geometry.

We calculate the methane excess adsorption in a similar way to experimental volumetric method: 1) by using helium adsorption in nanopores from GCMC simulation, we obtain the effective pore volume ${V}_{p}$. The details of effective pore volume calculation are shown in Supplementary Information. 2) Then, subtract the free gas occupied by ${V}_{p}$ from ${m}_{tot}$, which is calculated from GCMC simulations for given temperature and pressure conditions, as in Eq. (1). We will also compare the absolute adsorption from ${m}_{ex}$ and ${V}_{a}$ to that from ${m}_{ex}$ and ${\rho }_{a}$. In fact, using ${m}_{ex}$ and ${\rho }_{a}$ in Eq. (2) may bring significant error in the calculation of ${m}_{abs}$.

In addition, we will assess SDR model^31^ used in experiments to correct the excess adsorption to the absolute adsorption to provide insights into experimental fitting. Then, we will compare methane excess and absolute adsorptions in illite, montmorillonite and kaolinite nanopores.

The remainder of this paper is organized as follows. In section 2, we introduce the molecular simulation method and define the molecular models. In section 3, we first calibrate our GCMC simulation by comparing to experimental measurements and other molecular simulation works. Then, we compare different approaches to calculate absolute adsorption and assess SDR model. In section 4, we investigate methane adsorption in various clay-like slit pores with various pore sizes and temperatures. In section 5, we summarize key findings and implications.

## Molecular Model and Simulation

### Clay Minerals

In this subsection, we introduce the molecular configurations of illite, montmorillonite, and kaolinite nanopores. The clay atoms are fixed throughout our simulation.

#### Illite

**Figure 2:**
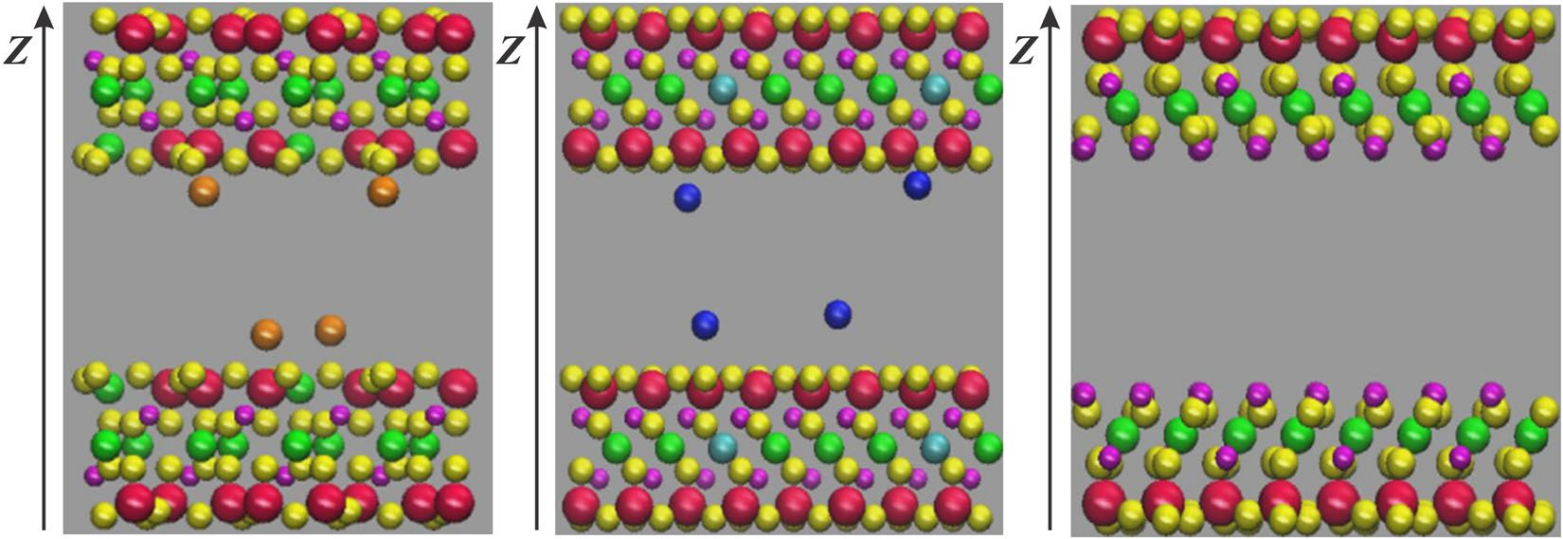
The schematic representation of clay nanopores (**a**) K-illite; (**b**) Na-montmorillonite; (**c**) kaolinite. Red spheres are Si atoms, green spheres are Al atoms, yellow spheres are O atoms, magenta spheres are H atoms, cyan spheres are Mg atoms, orange spheres are K^+^ ions and blue spheres are Na^+^ ions. The $z$ direction is perpendicular to the clay surfaces. The origin of the Cartesian coordinate is placed at the center of the simulation box.

Illite is one type of 2:1 clays consisting of two Si-O tetrahedral layers and one Al-O octahedral layer^47^. The original illite unit cell is Si_2_AlO_5_(OH), and the coordinate of each atom is from Pyrophyllite-1Tc powder diffraction^42,47,48^. The unit cell parameters are $a=0.51602$ nm, $b=0.89663$ nm, $c=0.93476$ nm, $\alpha =91.184^\circ$, $\beta =100.464^\circ$, and $\gamma =89.752^\circ$ ^49^. We duplicate the original unit cell in the ($-x$, $-y$, $-z$) direction and then the resulting structure is duplicated by displacing it a distance $a$ in the $x$ direction. The formula for the resulting 40-atom unit cell is Si_8_Al_4_O_20_(OH)_4_. The simulation cell consists of two clay sheets and each sheet contains 32 unit cells which is the result of replicating unit cells as $8\,\times \,4\,\times \,1$. They have the dimension of ${L}_{x}=4.128$ nm and ${L}_{y}=3.584$ nm in $x$ direction and $y$ direction, respectively. Two clay sheets are separated by a fixed distance to represent illite nanopore. The pore size $W$ is defined as the distance between the center of mass of oxygen atoms in the inner planes of the two sheets. In each 40-atom unit cell, one silica atom in tetrahedral layers is replaced by aluminum atom so that the clay sheet has a negative charge. The negative charge is neutralized by potassium ions distributed in pore space^36^. Unlike the clay atoms, these potassium ions are mobile in our simulation. With cation exchange, the unit cell formula is K(Si_7_Al)Al_4_O_20_(OH)_4_^33^. The resulting structure of illite nanopore is shown in Fig. 2a.

#### Montmorillonite

Montmorillonite is also 2:1 clay consisting of one Al-O layer and two Si-O layers. The neutral montmorillonite clay has a unit cell formula as Si_8_Al_4_O_20_(OH)_4_^50^. We adopted the atomic coordinates reported by Skipper *et al*.^21,51,52^. We use two clay sheets with 32 unit cells ($8\times 4\times 1$) in each to form montmorillonite clay nanopores with a patch of ${L}_{x}=4.224$ nm and ${L}_{y}=3.656$ nm in $x-y$ plane and thickness of 0.656 nm. Montmorillonite clay also has cation exchange capability with one aluminum atom replaced by magnesium atom in every 8 aluminum atoms in the octahedral sheet, and one silica atom replaced by aluminum atolm in every 32 silica atoms in tetrahedral sheet^52^. In our work, the negative charge is compensated by sodium ions. Similar to K-illite, these sodium ions are mobile in our simulation. The unit cell formula of Na-montmorillonite is Na_0.75_(Si_7.75_Al_0.25_)(Al_3.5_Mg_0.5_)O_20_(OH)_4_. The pore size $W$ is defined as the distance between the inner planes of the two sheets^21^. The resulting structure of montmorillonite nanopore is shown in Fig. 2b.

#### Kaolinite

Unlike illite and montmorillonite, kaolinite is one type of 1:1 clays composed of one Si-O tetrahedral and one Al-O octahedral sheet. X-ray diffraction analysis has shown that kaolinite has unit cell parameters as $a=0.5153$ nm, $b=0.8941$ nm, $c=0.7403$ nm, $\alpha ={91.692}^{\circ }$, $\beta ={104.86}^{\circ }$, and $\gamma ={89.822}^{\circ }$ ^53^. The formula of kaolinite unit cell is Al_4_Si_4_O_10_(OH)_8_^54^. We replicate the unit cell 8 times in $x$ direction and 4 times in $y$ direction. The resulting structure has a surface area as 4.1232 nm × 3.5768 nm. Two clay sheets are separated by a fixed distance to represent kaolinite nanopore and the Al-O plane is the inner plane of pore. The schematic representation of kaolinite nanopore is shown in Fig. 2c.

### Molecular Model and Force Fields

In our simulation, we use a single site model to describe methane and helium molecules. The TraPPE force field is used to represent the methane intermolecular interactions^55^. The interactions between methane/helium and clay atoms, and interlayer ions, and other methane/helium molecules are described by the pairwise-additive LJ 12-6 potentials:

4 $${u}_{LJ}({r}_{ij})=4{\varepsilon }_{ij}[{(\frac{{\sigma }_{ij}}{{r}_{ij}})}^{12}-{(\frac{{\sigma }_{ij}}{{r}_{ij}})}^{6}],$$

where ${r}_{ij}$, ${\varepsilon }_{ij}$, and ${\sigma }_{ij}$ are the separation, LJ well depth, and LJ size, respectively. The cross interactions between the unlike atoms and molecules, $i$ and $j$, are computed using the standard Lorentz-Berthelot combining rules^56^:

5 $${\sigma }_{ij}=({\sigma }_{ii}+{\sigma }_{jj})/2,$$

6 $${\varepsilon }_{ij}=\sqrt{{\varepsilon }_{ii}{\varepsilon }_{jj}}.$$

The $\varepsilon$ and $\sigma$ are 148.0 K and 0.373 nm, respectively, for methane molecules^55^, and 10.9 K and 0.264 nm, respectively, for helium molecules^57^. The interaction between interlayer ions and clay atoms are described by the sum of LJ and electrostatic interaction,

7 $$u({r}_{ij})={u}^{LJ}+{u}^{C}=4{\varepsilon }_{ij}[{(\frac{{\sigma }_{ij}}{{r}_{ij}})}^{12}-{(\frac{{\sigma }_{ij}}{{r}_{ij}})}^{6}]+\frac{{q}_{i}{q}_{j}}{4\pi {\varepsilon }_{0}{r}_{ij}},$$

in which ${q}_{i}$ is the partial charge of the site. We use CLAYFF force field^58^ to describe clay atoms and interlayer ions. The short-range LJ interactions are truncated at a distance of 1.07 nm without shift. Similar to our previous works^21,59^, to account for the long-range electrostatic interactions and the slab geometry that is periodic in $x-y$ plane and has a finite length in $z$ direction, we place a slab of vacuum in the simulation cell along the $z$ direction with a length much larger than ${L}_{x}$ or ${L}_{y}$ and use the standard three-dimensional *Ewald* summation with a correction term^60,61^.

### Simulations

Methane adsorption in various clay minerals are performed in the grand canonical ($\mu VT$) ensemble with simulation cell as a rectangular box with periodicity in $x$ and $y$ directions. The box sizes in $x$ and $y$ directions are represented by ${L}_{x}$ and ${L}_{y}$, respectively. The length in the $z$ direction is determined by the pore size of the clay and the vacuum^21^.

For simulations of methane molecules in clay nanopores, in each MC cycle, a trial random displacement is applied to randomly selected methane molecules and a methane molecule is randomly removed from or inserted into the simulation box at equal probability depending on the chemical potential of the methane reservoir outside^21^. The chemical potential of methane molecules is obtained from the Widom’s particle insertion method^62^ in canonical ($NVT$) ensemble without confinement. The bulk densities at given pressure and temperature are obtained from National Institute of Standards and Technology (NIST) Chemistry Webbook. The MC moves are implemented by the Metropolis algorithm^63^. The simulation consists of 0.2 million MC cycles per absorbate molecules for equilibrium and 0.8 million MC cycles per absorbate molecules for sampling density profiles.

### Effective Pore Volume

The calculation of effective pore volume is essential to the determination of the excess adsorption and consequently the absolute adsorption. In volumetric method, the effective pore volume ${V}_{p}$ is obtained by the helium adsorption^13,14^, based on the assumption that helium adsorption in nanopores is negligible and overall uptake is mainly dominated by pore filling. Zhang *et al*.^13^ obtained the void pore volume of different clay minerals from helium adsorption at pressure ranging from 6.9 to 150 bar with the average of five measurements. They postulated that the void volume remains constant over various temperatures. Similar to their experiment, in this work, we use helium adsorption at 333.15 K to calculate ${V}_{p}$ given as

8 $${V}_{p}=\frac{\langle {N}_{He}\rangle }{{N}_{A}{\rho }_{He,b}^{m}},$$

where $\langle {N}_{He}\rangle$ is the ensemble averaged number of helium molecules in given nanopores, ${N}_{A}$ is the Avogadro constant, and ${\rho }_{He,b}^{m}$ is the bulk molar density of helium at given pressures. We use five bulk pressures of 2, 4, 6, 8, and 10 MPa. The effective pore volume is the average of these five pressure conditions. We find that ${V}_{p}$ is independent of pressure and temperature. Due to the finite size of helium molecules, ${V}_{p}$ is less than simply multiplying the pore width $W$ and the surface area ${S}_{A}$. Chen *et al*.^38^ claimed that using $W\times {S}_{A}$ as pore volume may underestimate the excess adsorption. Details about the helium adsorption and pore volume calculations are presented in the Supplementary Information.

## Results and Discussion

### Calibration of GCMC Simulation

To calibrate our GCMC simulation, we compare the methane excess adsorption in illite, montmorillonite and kaolinite from our simulations to experimental measurements and other molecular simulation works^14,22,34,64^. Most of experimental data was reported as per unit mass of the adsorbent (i.e., mmol/g). In a recent work, Chen *et al*.^38^ claimed that to have a fair comparison between experiment and molecular simulation, one need to use per unit surface area of the adsorbent (i.e., mmol/m^2^). In experiment, SSA is generally obtained from the nitrogen adsorption^14^. In our simulation, we use the area of two *x* − y plane to describe SSA. The excess adsorption per surface area ${m}_{ex}$ is given as

9 $${m}_{ex}=\frac{\langle {N}_{{C}_{1}}\rangle /{N}_{A}-{V}_{p}{\rho }_{{C}_{1},b}^{m}}{2{S}_{A}}.$$

where $\langle {N}_{{C}_{1}}\rangle$ is the ensemble averaged number of methane molecules in the given nanopore and ${\rho }_{{C}_{1},b}^{m}$ is the bulk molar density of methane at a given pressure.

**Figure 3:**
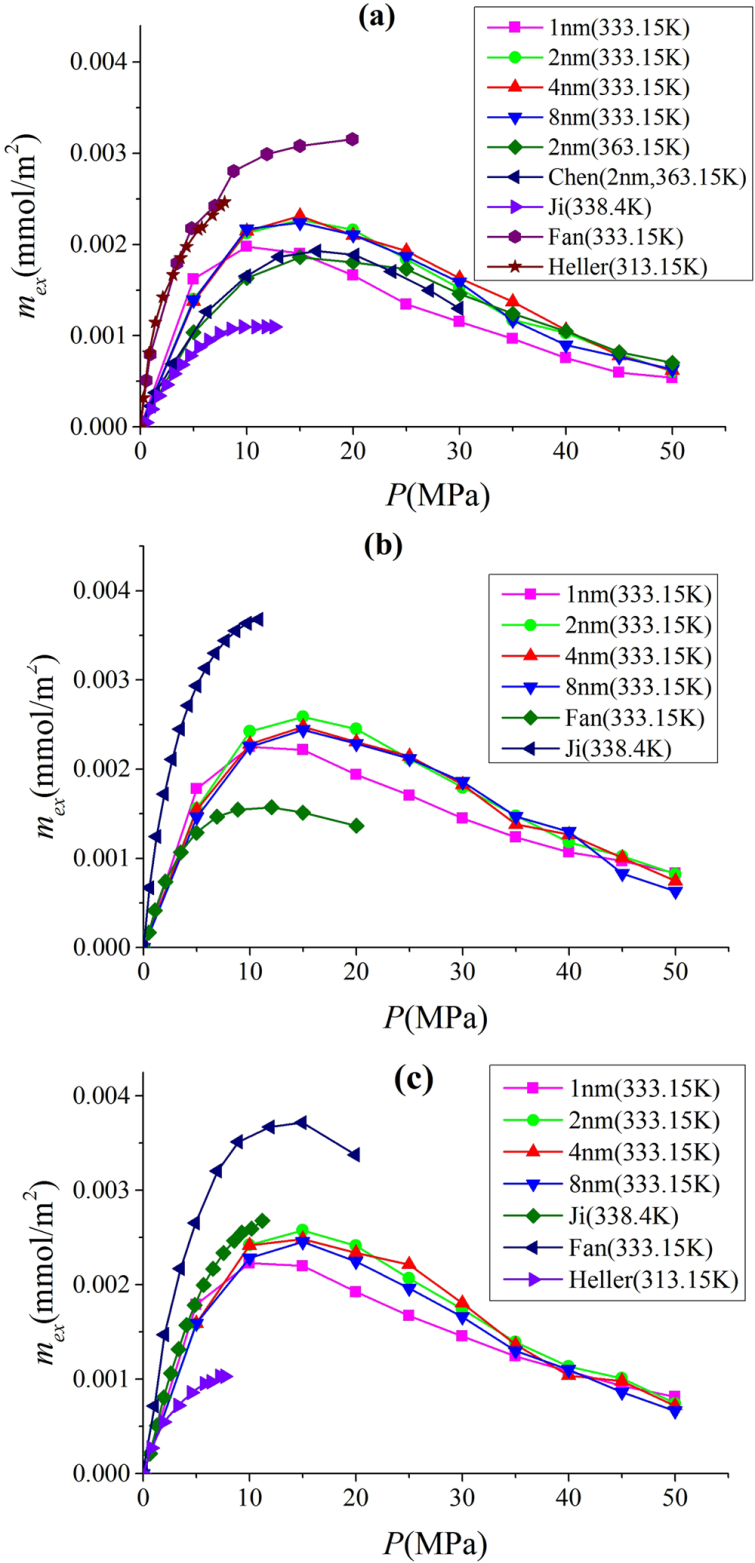
Excess adsorption from our GCMC simulations, experimental measurements by Ji *et al*.^14^, Fan *et al*.^64^, and Heller and Zoback^22^ as well as GCMC simulation by Chen *et al*.^34^ in (**a**) illite; (**b**) montmorillonite; (**c**) kaolinite.

In Fig. 3, we present ${m}_{ex}$ from our GCMC simulation and experimental data as well as other molecular simulation in various clay minerals. We use 41 m^2^/g^65^, 71.5 m^2^/g^14^, and 23.5 m^2^/g^66^ as SSA of illite, montmorillonite, and kaolinite from experiments, respectively. Heller and Zoback^22^ originally reported the absolute adsorption, assuming ${\rho }_{a}$ as saturated liquid density of 420 kg/m^3^ ^27^. We convert their absolute adsorption data to excess adsorption. Overall, our simulation result is in a general agreement with experimental data. There are some discrepancies probably due to following reasons: (1) SSA in experiment is obtained from low pressure nitrogen adsorption. Low pressure nitrogen adsorption is considered to explore the mesopores (pore size from 2 to 50 nm) in porous media^41^, while nitrogen molecules may not penetrate into micropores (pore size less than 2 nm). As a result, SSA might be underestimated^67^. In addition, SSA from nitrogen adsorption is affected by many subjective factors, such as crushed sample particle size^14^ and fitting range in the BET model^42^. (2) On the other hand, methane molecules can be adsorbed in both micropores and mesopores and surface adsorption in micropores can be significant^20^. (3) We simulated idealized condition as every pore is accessible to methane and helium molecules. However, in experiments, some of pores may be accessible to helium but not to methane molecules^38^. Chalmers *et al*.^41^ reported that shales can have large amount of nanopores with diameters down to 0.3 nm. While helium molecules may adsorb in such small nanopores, methane may not penetrate into. Considering these factors, our simulation is in a reasonable agreement with experimental data. In addition, our simulation shows excellent agreement with GCMC simulation by Chen *et al*.^34^ in 2 nm illite pores at 363.15 K, which is obtained from their total adsorption with ${V}_{p}$ from our calculations.

We observe that when the pore size is larger than 2 nm, ${m}_{ex}$ becomes insensitive to $W$. In other GCMC simulation works by Chen *et al*.^34^ and Xiong *et al*.^35^, the excess adsorption per SSA decreases with $W$. Interestingly, in another work, Chen and his workers found that excess adsorption per specific surface area of CO_2_ and N_2_ does not change with pore size when $W\ge 2$ nm^36^. In fact, the excess adsorption is very sensitive to the calculation of ${V}_{p}$ ^38^. Do *et al*.^37^ have shown that a small change in ${V}_{p}$ can have significant effect on excess adsorption. Our calculation reveals that if ${V}_{p}$ is obtained from helium adsorption, the excess adsorption becomes similar when $W\ge 2$ nm. In entire pressure range, unlike other simulation works^33,38^, the excess adsorption is always positive. As a result, the calculation of the adsorbed layer density from the intercept of excess adsorption^33^ may not be applicable.

### Density profiles

**Figure 4:**
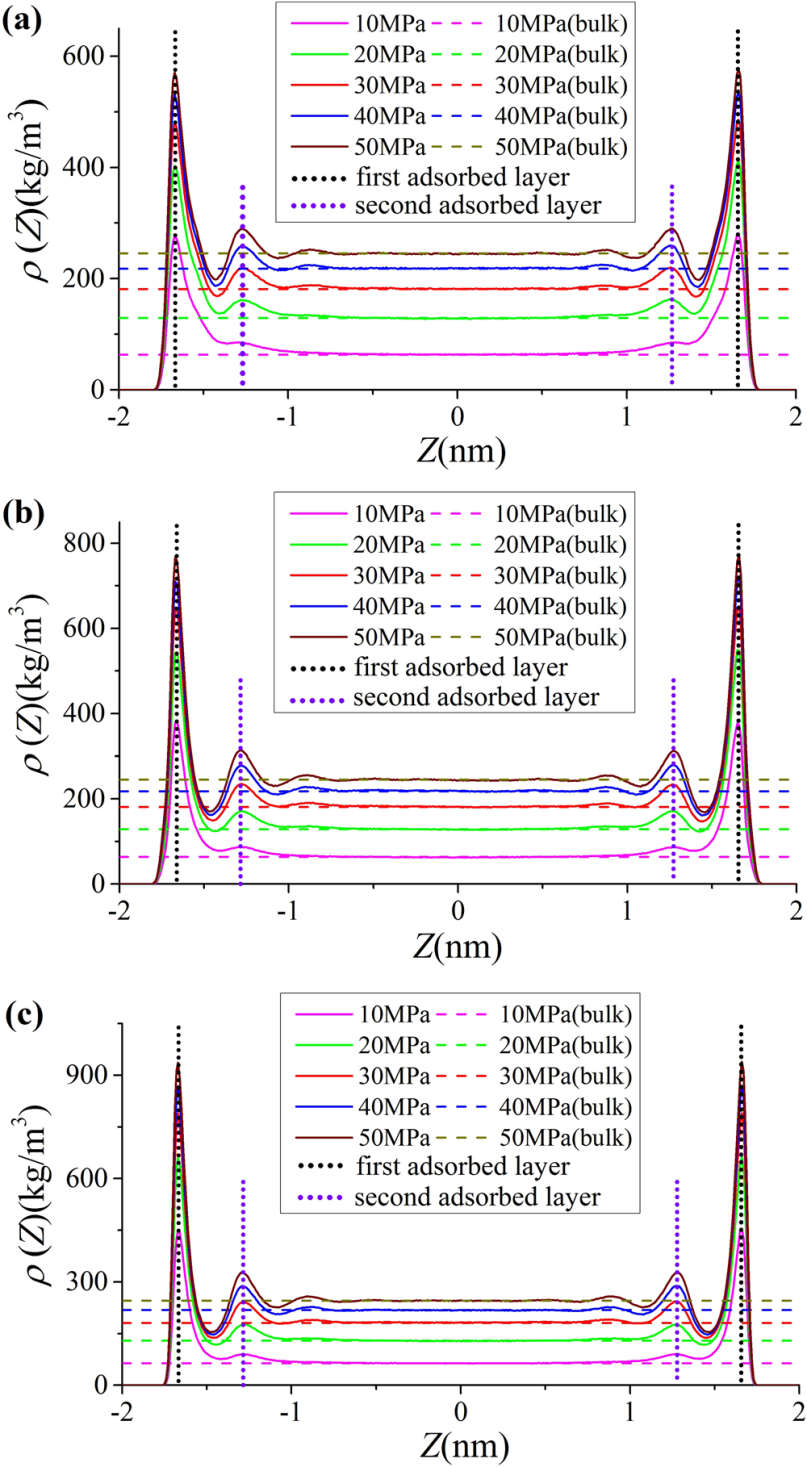
Methane density profiles in (**a**) illite; (**b**) montmorillonite; (**c**) kaolinite nanopores of $W=4$ nm at 333.15 K with varying bulk pressures. The solid lines are density distributions from GCMC simulation, dashed lines are the guidelines for bulk densities from NIST Chemistry Webbook, and the dotted lines are the guidelines for the first and second adsorption layers.

To better understand methane adsorption behavior in clay nanopores, in Fig. 4, we present the methane density distributions in 4-nm illite, montmorillonite, and kaolinite nanopores at various bulk pressures and 333.15 K. For all bulk pressure conditions, methane forms a strong adsorption layer and the density in the middle of the pores approaches bulk. In general, as bulk pressure increases, the adsorption layer density increases. As a result, using a constant adsorbed layer density^22^ may not be justifiable to predict the absolute adsorption in Eq. (2). At high pressure conditions, e.g. bulk pressure of 50 MPa, methane may form a weak second adsorption layer. However, as we will discuss later, such second adsorption layer may be “averaged out” by the saddle point between the first and second adsorption layers and the averaged density in the second adsorption layer becomes comparable to the bulk density. At relat﻿ivel﻿y lower pressures, e.g. bulk pressure of 10 MPa, after the first adsorption layer, the density is slightly higher than the bulk density, which may indicate a transition zone in density distributions^68^. Such transition zone may significantly affect absolute adsorption calculation.

**Figure 5:**
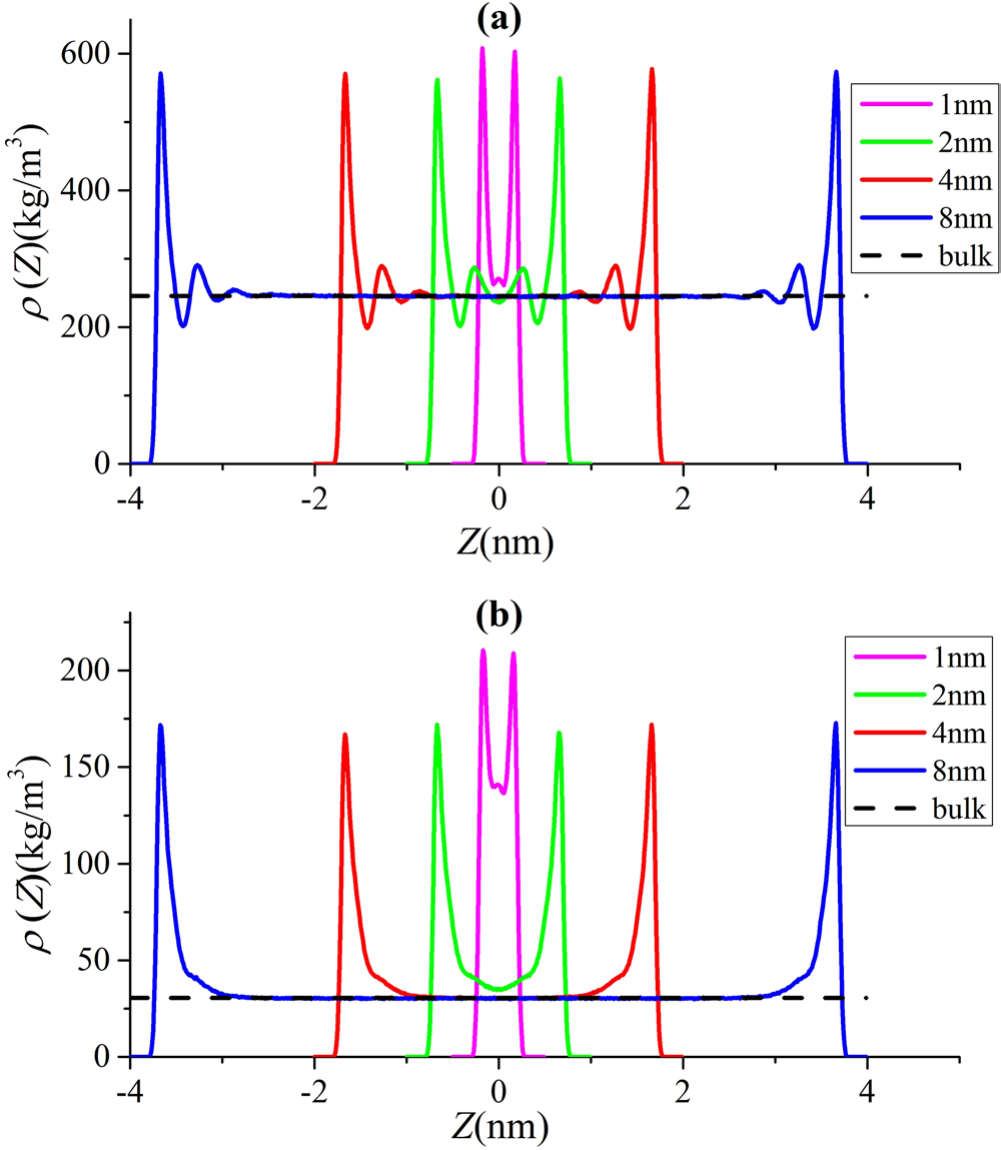
Methane density distributions in illite nanopores of varying pore sizes at 333.15 K and (**a**) $P=50$ MPa; (**b**) $P=5$ MPa. The solid lines represent the density profiles from GCMC simulations and dashed lines are guidelines for ${\rho }_{b}$ from NIST Chemistry Webbook.

To understand the effect of pore size on methane adsorption, we present the density distributions in illite nanopores of varying pore widths at 333.15 K in Fig. 5. At $P=50$ MPa, except $W=1$ nm, methane can form two adsorption layers on the surface. Within 1 nm pores, due to limited pore space, methane can form only one adsorption layer on the surface. When $W\ge 4$ nm, the density in the middle of the pores approaches bulk, while methane shows varying density distributions in the pores for $W\le 2$ nm. As a result, for $W\le 2$ nm, the adsorption model proposed in Fig. 1 may become inapplicable. On the other hand, at $P=5$ MPa, methane can form one adsorption layer on the surface and transition zone is observed for 4 and 8 nm pores. It has been debated in the past on whether methane adsorption mechanism in shale is single layer adsorption or micropore filling^11,69–73^. Our results indicate that adsorption mechanism varies by pore size. In mesopores defined by IUPAC^74^, methane can form strong adsorption layer on the surface and density in the middle of the approaches bulk density. In micropores, due to limited pore space, methane can only form adsorption layer on the surface. It means that there is no free gas region in micropores, and absolute adsorption capacity is the same as the total methane capacity. These results agree with the past works^4,75,76^ that under in-situ shale reservoir condition, methane fills micropores most, and monolayer adsorption occurs in larger pores.

### Absolute adsorption

In this subsection, we use methane adsorption in illite at 333.15 K as an example to compare different approaches to calculate the absolute adsorption.

**Figure 6:**
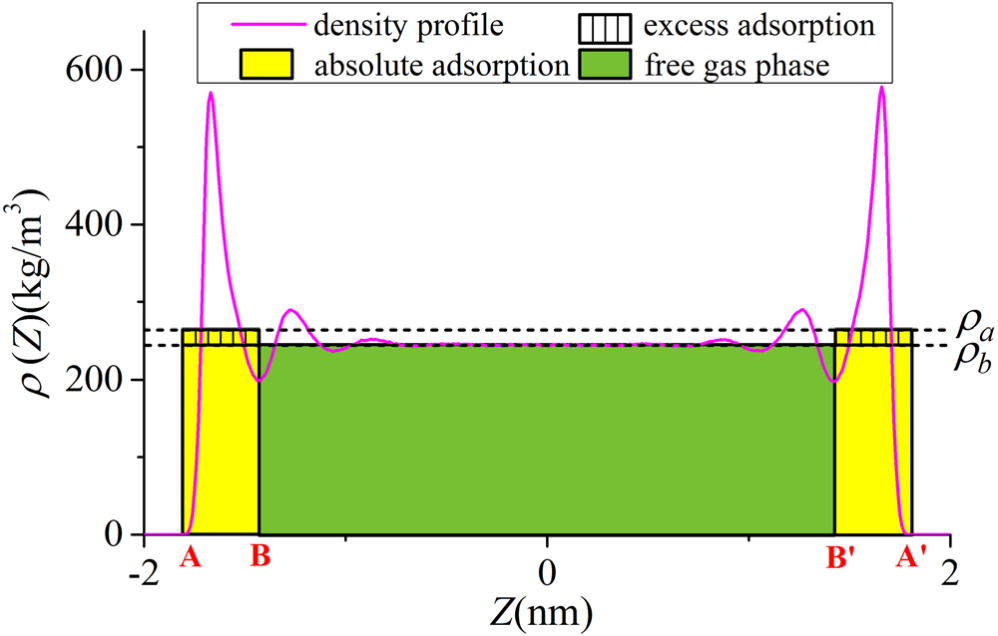
The schematic representation of adsorbed and free gas phases for methane adsorption in illite nanopore of $W=4$ nm at 333.15 K and 50 MPa. The heights of adsorbed and free gas phases are depicted from ${\rho }_{a}={\int }_{A}^{B}\rho (z)dz/{z}_{AB}$ and ${\rho }_{b}$ from NIST Chemistry Webbook, respectively.

**Figure 7:**
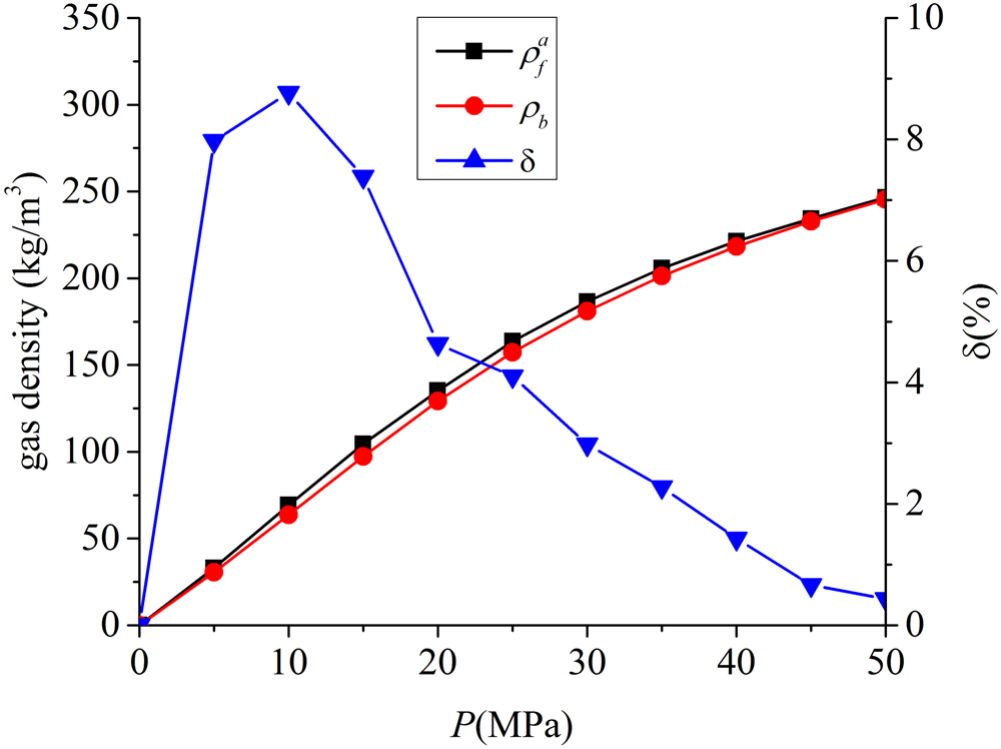
Comparison of ${\rho }_{f}^{a}={\int }_{B}^{{B}^{^{\prime} }}\rho (z)dz/{z}_{B{B}^{^{\prime} }}$ from density distributions and ${\rho }_{b}$ from NIST Chemistry Webbook and the variance $\delta =({\rho }_{f}^{a}-{\rho }_{b})/{\rho }_{b}$ in illite nanopore of $W=4$ nm at 333.15 K.

**Figure 8:**
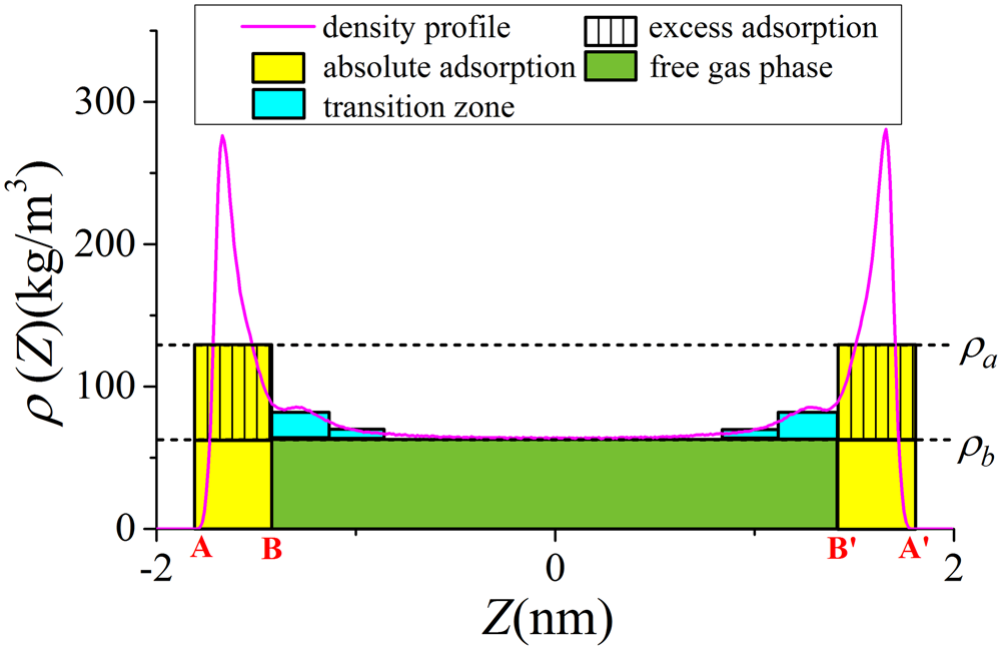
The schematic representation of transition zone in illite nanopores of $W=4$ nm at 333.15 K and 10 MPa. The heights of adsorbed and free gas phases are depicted from ${\rho }_{a}={\int }_{A}^{B}\rho (z)dz/{z}_{AB}$ and ${\rho }_{b}$ from NIST Chemistry Webbook, respectively.

As shown in Figs 4 and 5, in micropores, the surface adsorption dominates and the density in the middle of pore does not approach bulk. In mesopores, however, methane can have a strong adsorption layer on the surface and the density in the middle of the pores approaches bulk. Based on such adsorption mechanism, in mesopores, one can define the adsorbed and free gas phases, respectively, as shown in Fig. 6. In addition, ${V}_{p}$ obtained from the helium adsorption is depicted as the region between Point A and A’, ${z}_{AA\text{'}}$, which is defined as ${z}_{AA\text{'}}={V}_{p}/{S}_{A}$. The adsorbed phase is defined as the region between the Point A and B, which is the saddle point between the first and second adsorption layers. The width of AB in the $z$ direction, ${z}_{AB}$, is around 0.38 nm, similar to the LJ diameter of methane molecules, while methane adsorption is considered to be single-layered. Previous theoretical calculations on LJ fluid adsorption in nanopores reveals that the width of adsorption layer is equal to the diameter of absorbate molecules^77^. In addition, Didar and Akkutlu^68^ pointed out that the width of adsorbed phase is equal to the molecular diameter. As shown in Fig. 4, at high pressure conditions, the Point B does not change with pressure. We use the Point B at $P=50$ MPa as the boundary for adsorbed phase and assume a constant adsorbed phase volume ${V}_{a}=2{S}_{A}{z}_{AB}$ for various pressure conditions. The adsorbed phase covers the strong surface adsorption layer, where the adsorbed phase density ${\rho }_{a}={\int }_{A}^{B}\rho (z)dz/{z}_{AB}$ can be higher than ${\rho }_{b}$. The amount in the adsorbed phase is the absolute adsorption, ${m}_{abs}={\rho }_{a}{V}_{a}$. In other words, once the adsorbed phase is defined, the absolute adsorption can be readily obtained from density distributions. The free gas phase is between Point B and B′, which covers the weak second adsorption layer. We depict the average density of free gas phase ${\rho }_{f}^{a}={\int }_{B}^{B\text{'}}\rho (z)dz/{z}_{BB\text{'}}$ (${z}_{BB\text{'}}$ is the distance between Point B and B′) and ${\rho }_{b}$ from NIST Chemistry Webbook in Fig. 7. The difference between ${\rho }_{f}^{a}$ and ${\rho }_{b}$ becomes negligible at high pressures. At 50 MPa, the variance $\delta =({\rho }_{f}^{a}-{\rho }_{b})/{\rho }_{b}$ is around 0.5%. At higher pressures up to 80 MPa, $\delta$ is less than 0.5% (shown in Supplementary Information). It shows that our classification agrees excellently with the adsorption model shown in Fig. 1. At low pressure conditions, there is some discrepancy between ${\rho }_{f}^{a}$ and ${\rho }_{b}$, e.g. $\delta$ around 9% at 10 MPa. It is due to the presence of transition zone^68^ as shown in Fig. 8. To have a better fit with the adsorption model shown in Fig. 1, the transition zone ‘should’ have been included in the adsorbed phase. However, Didar and Akkutlu^68^ argued that the transition zone is less influenced by the wall and may not behave as adsorbed phase. Due to transition zone, ${\rho }_{f}^{a}$ is higher than ${\rho }_{b}$.

**Figure 9:**
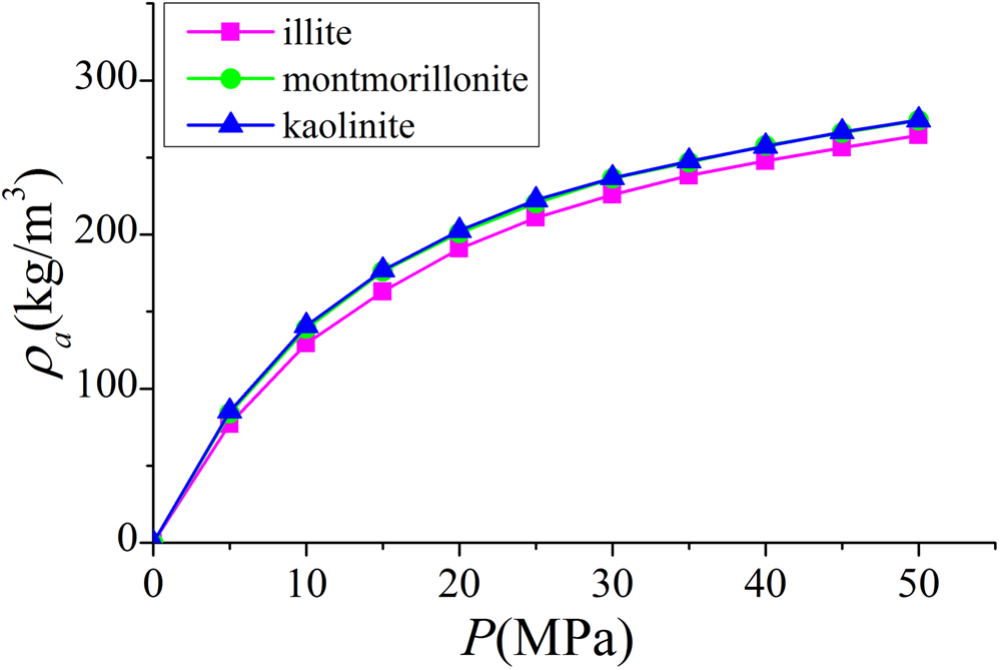
${\rho }_{a}$ in illite, montmorillonite and kaolinite nanopores of $W=4$ nm at 333.15 K.

**Figure 10:**
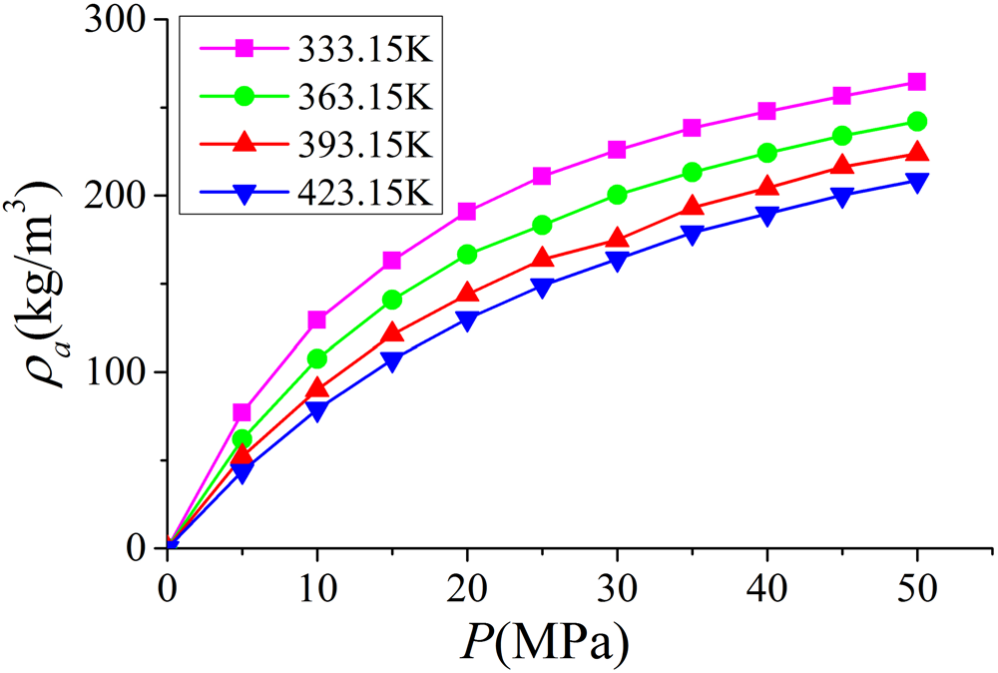
${\rho }_{a}$ in illite nanopores of $W=4$ nm at different temperatures.

**Figure 11:**
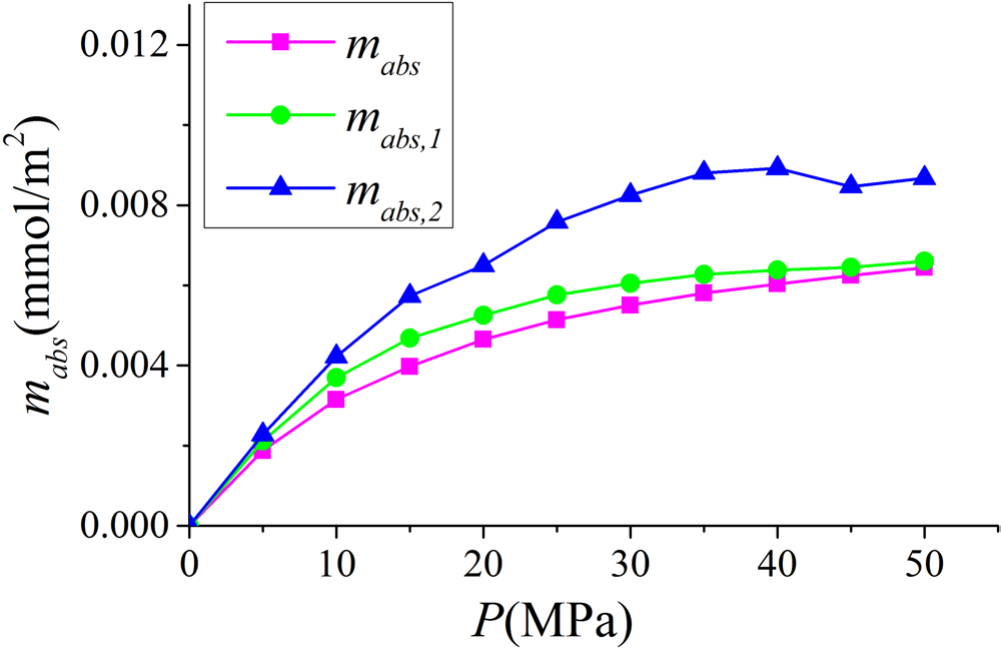
Absolute adsorption in illite nanopores of $W=4$ nm at 333.15 K. ${m}_{abs}={\rho }_{a}{V}_{a}$ is based on density distribution; ${m}_{abs,1}={m}_{ex}+{\rho }_{b}{V}_{a}$ is obtained from ${m}_{ex}$ and ${V}_{a}$; ${m}_{abs,2}={m}_{ex}/(1-{\rho }_{b}/{\rho }_{a})$ is obtained from ${m}_{ex}$ and ${\rho }_{a}$.

In Fig. 9, we present the adsorbed phase density ${\rho }_{a}$ in various clay nanopores of 4 nm at 333.15 K. ${\rho }_{a}$ continuously increases with pressure up to 50 MPa. With a constant adsorbed phase volume ${V}_{a}$, absolute adsorption continuously increases with pressure (pressure up to 80 MPa shown in Supplementary Information). For different clay minerals, ${\rho }_{a}$ is similar. We also present the effect of temperature on ${\rho }_{a}$ in Fig. 10. As temperature increases, ${\rho }_{a}$ decreases due to weaker fluid-surface interactions.With the definition of adsorbed and free gas phases, in Fig. 11, we present the absolute adsorption based on ${m}_{ex}$ and ${\rho }_{a}$, and ${m}_{ex}$ and ${V}_{a}$ as in Eqs (2) and (3), respectively. We also depict the absolute adsorption given as ${m}_{abs}={\rho }_{a}{V}_{a}$, which is from the density distributions. It shows that the absolute adsorption from ${m}_{ex}$ and ${\rho }_{a}$ is overestimated (${m}_{abs,2}={m}_{ex}/(1-{\rho }_{b}/{\rho }_{a})$ in Fig. 11), especially at low pressure conditions. It is due to the presence of transition zone. The transition zone contributes to ${m}_{ex}$, while ${\rho }_{a}$ only takes into account the excess adsorption amount in adsorbed phase. On the other hand, the absolute adsorption from ${m}_{ex}$ and ${V}_{a}$ (${m}_{abs,1}={m}_{ex}+{\rho }_{b}{V}_{a}$ in Fig. 11) shows an excellent agreement with ${m}_{abs}$. The agreement is better at higher pressures as shown in Supplementary Information. At low pressure conditions, due the presence of transition zone, there is a small discrepancy and the adsorption model shown in Fig. 1 is less suitable to describe the adsorption behavior. Using both varying ${\rho }_{a}$ and ${V}_{a}$ would make the adsorption model a pure fitting without rigorous physical foundation. As pressure increases, the deficiency becomes negligible.

Based on Eq. (3), difference between ${m}_{abs}$ and ${m}_{abs,1}$, ${\rm{\Delta }}{m}_{1}$ is

10 $${\rm{\Delta }}{m}_{1}={m}_{abs,1}-{m}_{abs}={m}_{ex}-({\rho }_{a}-{\rho }_{b}){V}_{a},$$

while according to Eq. (2), difference between ${m}_{abs}$ and ${m}_{abs,2}$, ${\rm{\Delta }}{m}_{2}$ is

11 $${\rm{\Delta }}{m}_{2}={m}_{abs,2}-{m}_{abs}=\frac{{\rho }_{a}}{{\rho }_{a}-{\rho }_{b}}\cdot [{m}_{ex}-({\rho }_{a}-{\rho }_{b}){V}_{a}].$$

**Figure 12:**
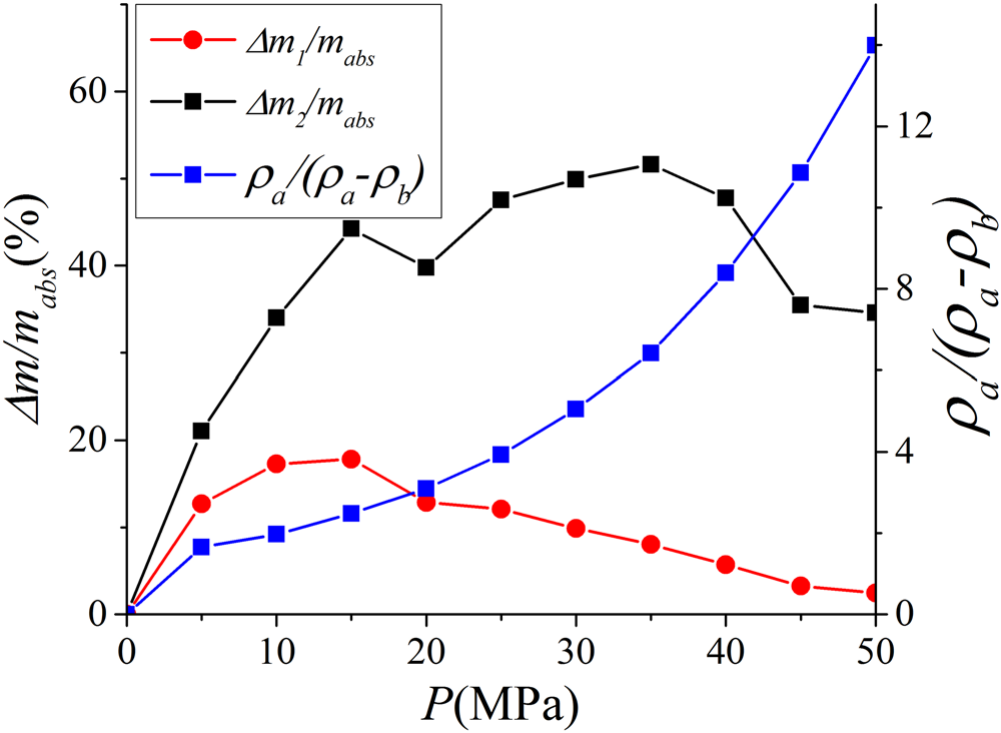
The variances of absolute adsorption ${m}_{abs,1}$ obtained by ${m}_{ex}$ and ${V}_{a}$; ${m}_{abs,2}$ obtained by ${m}_{ex}$ and ${\rho }_{a}$ from ${m}_{abs}={\rho }_{a}{V}_{a}$. We also present ${\rho }_{a}/({\rho }_{a}-{\rho }_{b})$ versus pressure.

As shown in Fig. 12, ${\rm{\Delta }}{m}_{1}/{m}_{abs}$ continuously decreases with pressure beyond 15 MPa. Especially at high pressure, ${\rm{\Delta }}{m}_{1}/{m}_{abs}$ is only around 3%. On the other hand, ${\rm{\Delta }}{m}_{2}/{m}_{abs}$ is around 30% at high pressure. The difference between Eqs (10) and (11) is the coefficient ${\rho }_{a}/({\rho }_{a}-{\rho }_{b})$, which continuously increases with pressure. As a result, ${m}_{abs,1}$ obtained from ${m}_{ex}$ and ${V}_{a}$ is more accurate than ${m}_{abs,2}$ from ${m}_{ex}$ and ${\rho }_{a}$ for a given adsorbed phase.

**Figure 13:**
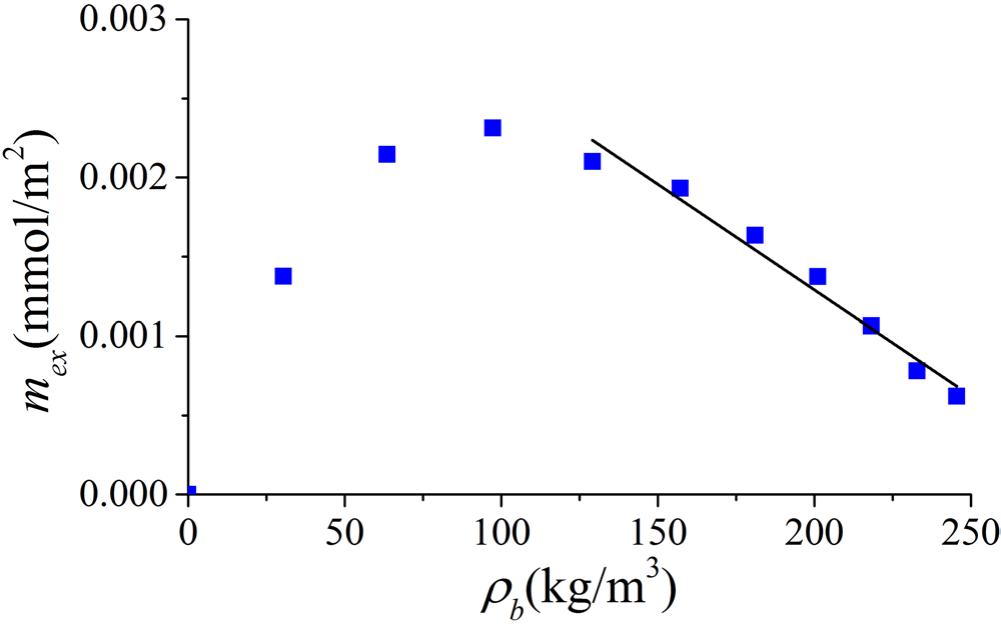
Excess adsorption versus ${\rho }_{b}$ in illite nanopore of $W=4$ nm at 333.15 K. The solid line is linear fitting at high pressures to calculate ${V}_{a}$ from the slope method by Zhang *et al*.^33^.

**Figure 14:**
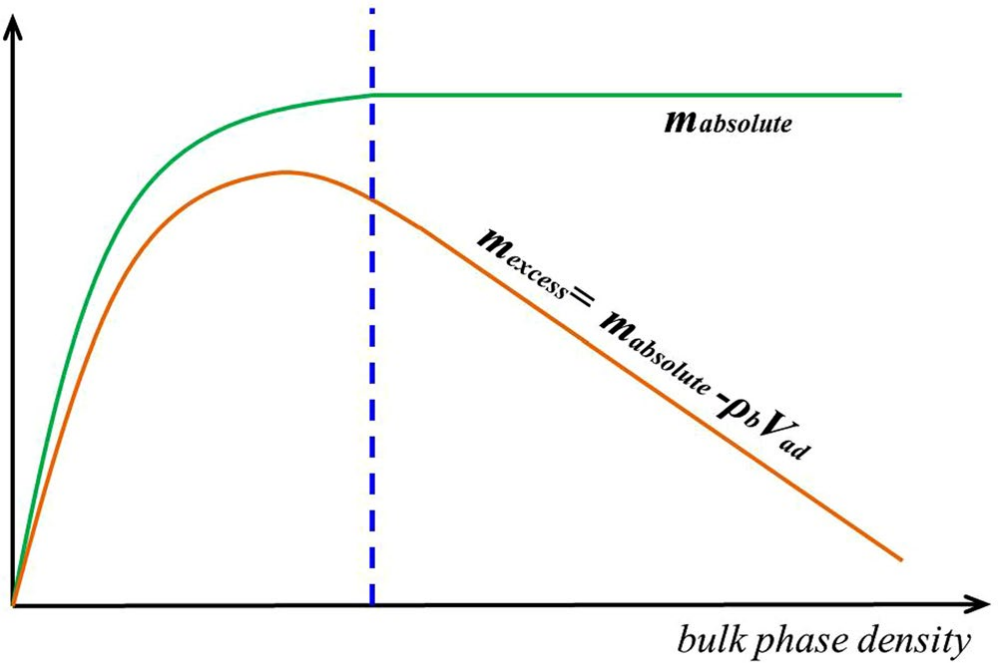
Ideal excess and absolute adsorption isotherms suitable for determining ${V}_{a}$ from the slope method by Zhang *et al*.^33^.

Zhang *et al*.^33^ used the slope of the excess adsorption with respect to the corresponding bulk density as adsorbed phase volume at high pressure conditions. Based on the slope method as depicted in Fig. 13, the calculated ${V}_{a}$ is around 6.315 nm^3^ for 4 nm-illite at 333.15 K. This results in a width of adsorbed phase of 0.213 nm, much less than methane diameter. The slope method is considered to be valid only when the absolute adsorption becomes constant as depicted in Fig. 14. However, as shown in Fig. 11 and Figure SI.B1, absolute adsorption keeps increasing with pressure. As a result, the slope method may not be applicable.

### Assessment of Supercritical Dubinin-Radushkevich (SDR) Fitting Method

Recently, a number of fitting methods, such as Langmuir^78^, SDR^79^, and Ono-Kondo (OK)^80^ models have been used to fit the excess adsorption and then correct to the absolute adsorption. Among them, SDR model based on pore-filling theory, is a popular choice to obtain the sorption capacity of porous materials^31^. The SDR model for absolute adsorption is given as^79^,

12 $${m}_{abs}^{SDR}={m}_{abs,\,\max }^{SDR}\,\exp \,\{-C{[\mathrm{ln}({\rho }_{a,\max }^{SDR}/{\rho }_{b})RT]}^{2}\},$$

in which ${m}_{abs}^{SDR}$ is the absolute adsorption of a given pressure, ${m}_{abs,\,\max }^{SDR}$ is the maximum absolute adsorption, ${\rho }_{a,\,\max }^{SDR}$ is the maximum adsorbed phase density, $C$ is the interaction constant, $R$ is the gas constant, and $T$ is the absolute temperature. Note that ${m}_{abs}^{SDR}$, ${m}_{abs,\,\max }^{SDR}$, and ${\rho }_{a,\,\max }^{SDR}$ are all from the SDR fitting, not from density distributions. To fit with ${m}_{ex}$, two options have been used for the modification^4^, either (*) from the constant adsorbed phase volume ${V}_{a}^{SDR{,}^{\ast }}$,

13 $${m}_{ex}={m}_{abs}^{SDR}-{\rho }_{b}{V}_{a}^{SDR{,}^{\ast }},$$

or (**) from the constant adsorbed phase density ${\rho }_{a}^{SDR{,}^{\ast \ast }}$,

14 $${m}_{ex}={m}_{abs}^{SDR}(1-\frac{{\rho }_{b}}{{\rho }_{a}^{SDR{,}^{\ast \ast }}}).$$

In the following subsections, we will assess SDR models assuming constant adsorbed phase volume or density by fitting with our ${m}_{ex}$ at a given temperature.

#### SDR Fitting with Constant Adsorbed Phase Volume

With constant ${V}_{a}^{SDR{,}^{\ast }}$, ${m}_{abs,\,\max }^{SDR{,}^{\ast }}$ can be expressed in terms of ${m}_{abs,\,\max }^{SDR}={\rho }_{a,\,\max }^{SDR{,}^{\ast }}{V}_{a}^{SDR{,}^{\ast }}$ and Eq. (12) can be rewritten as

15 $${m}_{abs}^{SDR{,}^{\ast }}={\rho }_{a,\,\max }^{SDR{,}^{\ast }}{V}_{a}^{SDR{,}^{\ast }}\exp \{-{C}^{\ast }{[\mathrm{ln}({\rho }_{a,\max }^{SDR{,}^{\ast }}/{\rho }_{b})RT]}^{2}\},$$

and Eq. (13) can be expressed as

16 $${m}_{ex}={\rho }_{a,\,\max }^{SDR{,}^{\ast }}{V}_{a}^{SDR{,}^{\ast }}\,\exp \,\{-{C}^{\ast }{[\mathrm{ln}({\rho }_{a,\max }^{SDR{,}^{\ast }}/{\rho }_{b})RT]}^{2}\}-{\rho }_{b}{V}_{a}^{SDR{,}^{\ast }}.$$

Comparing to the adsorption model shown in Fig. 1, the adsorbed phase density ${\rho }_{a}^{SDR{,}^{\ast }}$ for a given pressure can be calculated from

17 $${\rho }_{a}^{SDR{,}^{\ast }}={\rho }_{a,\,\max }^{SDR{,}^{\ast }}\exp \{-{C}^{\ast }{[\mathrm{ln}({\rho }_{a,\max }^{SDR{,}^{\ast }}/{\rho }_{b})RT]}^{2}\}.$$

**Figure 15:**
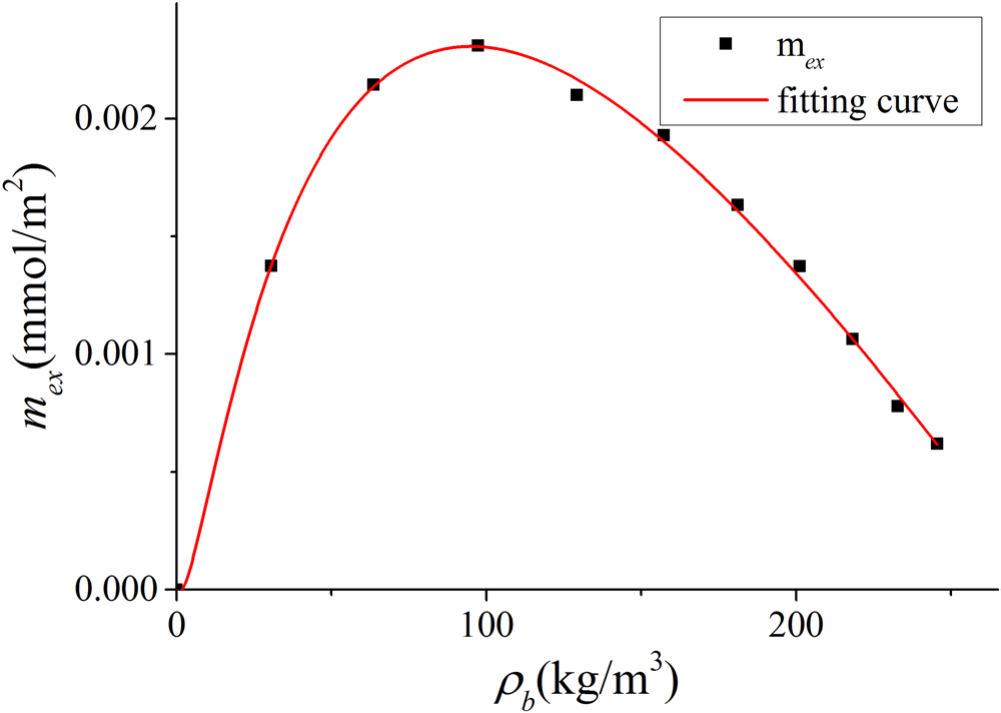
SDR Fitting with constant ${V}_{a}^{SDR{,}^{\ast }}$ to the excess adsorption in illite nanopores of $W=4$ nm at 333.15 K with ${R}^{2}=0.9975$.

Our excess adsorption is fitted by the least-square method with all free parameters in Eq. (16) varying over the following ranges: $0 < {\rho }_{a,\,\max }^{SDR{,}^{\ast }} < 500$ kg/m^3^, ${V}_{a}^{SDR{,}^{\ast }} > 0$ nm^3^, and $0 < {C}^{\ast } < 0.05$ mol^2^/kJ^2^ ^31^. Excess adsorption in illite nanopores of $W=4$ nm at 333.15 K is used for SDR fitting with constant ${V}_{a}^{SDR{,}^{\ast }}$ as in Fig. 15. The fitting parameters are ${\rho }_{a,\max }^{SDR{,}^{\ast }}=\mathrm{28}0.65067$ kg/m^3^, ${V}_{a}^{SDR{,}^{\ast }}={\rm{8.56702}}$ nm^3^, and ${C}^{\ast }=0.02564$ mol^2^/kJ^2^, respectively.

**Figure 16:**
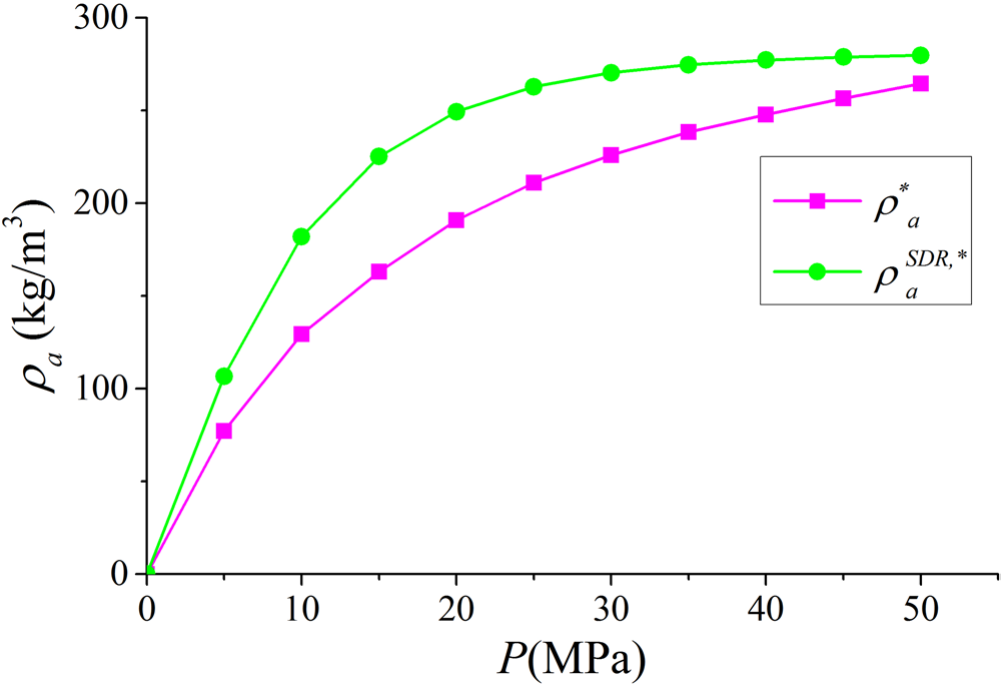
${\rho }_{a}^{\ast }$ from the integration of density distributions within adsorbed phase with volume ${V}_{a}^{SDR{,}^{\ast }}$ and ${\rho }_{a}^{SDR{,}^{\ast }}$ from SDR fitting as shown in Eq. (17).

The corresponding adsorbed phase width is around 0.29 nm, which is less than the methane molecule diameter. With the fitted ${V}_{a}^{SDR{,}^{\ast }}$, based on adsorption model shown in Fig. 1, adsorbed phase density ${\rho }_{a}^{\ast }$ can be obtained from the integration of density distributions within adsorbed phase as ${\rho }_{a}^{\ast }={\int }_{A}^{{B}^{\ast }}\rho (z)dz/{z}_{A{B}^{\ast }}$, in which ${B}^{\ast }$ is the boundary of adsorbed phase with volume ${V}_{a}^{SDR{,}^{\ast }}$ and ${z}_{A{B}^{\ast }}={V}_{a}^{SDR{,}^{\ast }}/2{S}_{A}$ represents the width of the corresponding adsorbed phase. In Fig. 16, we depict ${\rho }_{a}^{\ast }$ and ${\rho }_{a}^{SDR{,}^{\ast }}$ from Eq. (17). For all pressure conditions, ${\rho }_{a}^{SDR{,}^{\ast }}$ is higher than ${\rho }_{a}^{\ast }$. The difference first increases with pressure, then decreases.

**Figure 17:**
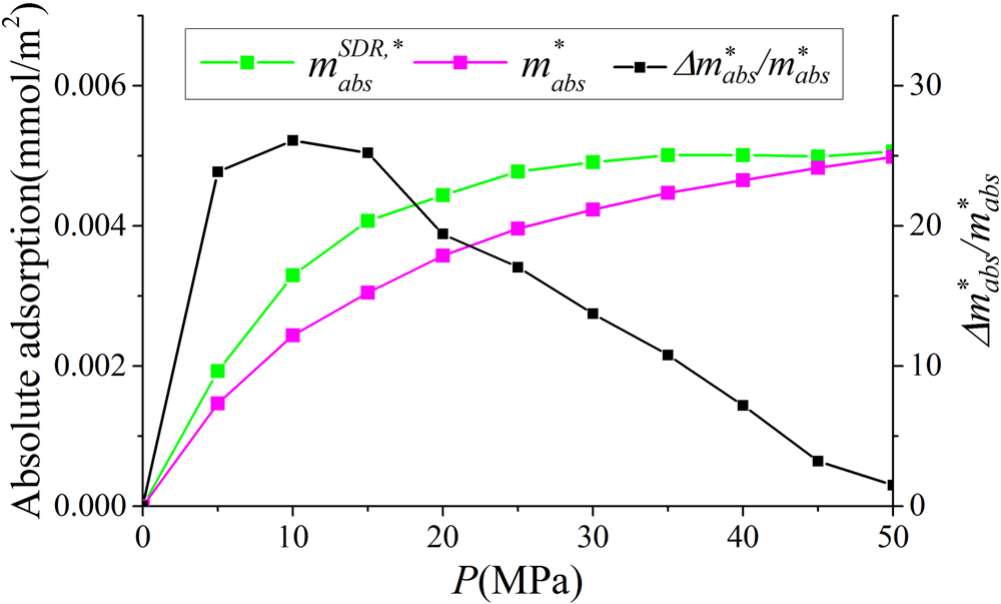
Absolute adsorption ${m}_{abs}^{\ast }={\rho }_{a}^{\ast }{V}_{a}^{SDR{,}^{\ast }}$ and ${m}_{abs}^{SDR{,}^{\ast }}={m}_{ex}+{\rho }_{b}{V}_{a}^{SDR{,}^{\ast }}$ from SDR model with constant ${V}_{a}^{SDR{,}^{\ast }}$ as well as the variance of absolute adsorption $\Delta {m}_{abs}^{\ast }/{m}_{abs}^{\ast }$.

We depict the absolute adsorption ${m}_{abs}^{\ast }={\rho }_{a}^{\ast }{V}_{a}^{SDR{,}^{\ast }}$ from density distributions and ${m}_{abs}^{SDR{,}^{\ast }}={m}_{ex}+{\rho }_{b}{V}_{a}^{SDR{,}^{\ast }}$ from the SDR model with constant ${V}_{a}^{SDR{,}^{\ast }}$ in Fig. 17. Similar to Fig. 11, there is some discrepancy when pressure is relatively low and agreement becomes better at high pressure conditions. The difference is due to the presence of transition zone. In Fig. 17, We also present the variance of absolute adsorption, $\Delta {m}_{abs}^{\ast }/{m}_{abs}^{\ast }=({m}_{abs}^{SDR{,}^{\ast }}-{m}_{abs}^{\ast })/{m}_{abs}^{\ast }$. Comparing to our model as shown in Fig. 12, the variance is larger. It is because in SDR model with constant ${V}_{a}^{SDR{,}^{\ast }}$, more transition zone is included in the free gas phase.

#### SDR Fitting with Constant Adsorbed Phase Density

With constant ${\rho }_{a}^{SDR{,}^{\ast \ast }}$, ${\rho }_{a,\,\max }^{SDR{,}^{\ast \ast }}$ is the same as ${\rho }_{a}^{SDR{,}^{\ast \ast }}$. ${m}_{abs,\,\max }^{SDR{,}^{\ast \ast }}$ can be expressed in terms of ${m}_{abs,\max }^{SDR{,}^{\ast \ast }}={\rho }_{a}^{SDR{,}^{\ast \ast }}{V}_{a,\max }^{SDR{,}^{\ast \ast }}$ and Eq. (12) can be rewritten as

18 $${m}_{abs}^{SDR{,}^{\ast \ast }}={\rho }_{a}^{SDR{,}^{\ast \ast }}{V}_{a,\max }^{SDR{,}^{\ast \ast }}\,\exp \,\{-{C}^{\ast \ast }{[\mathrm{ln}({\rho }_{a}^{SDR{,}^{\ast \ast }}/{\rho }_{b})RT]}^{2}\},$$

and Eq. (14) can be expressed as

19 $${m}_{ex}={\rho }_{a}^{SDR{,}^{\ast \ast }}{V}_{a,\max }^{SDR{,}^{\ast \ast }}\exp \{-{C}^{\ast \ast }{[\mathrm{ln}({\rho }_{a}^{SDR{,}^{\ast \ast }}/{\rho }_{b})RT]}^{2}\}(1-\frac{{\rho }_{b}}{{\rho }_{a}^{SDR{,}^{\ast \ast }}})$$

Comparing to the adsorption model shown in Fig. 1, the adsorbed phase volume ${V}_{a}^{SDR{,}^{\ast \ast }}$ for a given pressure can be calculated from

20 $${V}_{a}^{SDR{,}^{\ast \ast }}={V}_{a,\max }^{SDR{,}^{\ast \ast }}\exp \{-{C}^{\ast \ast }{[\mathrm{ln}({\rho }_{a}^{SDR{,}^{\ast \ast }}/{\rho }_{b})RT]}^{2}\}.$$

**Figure 18:**
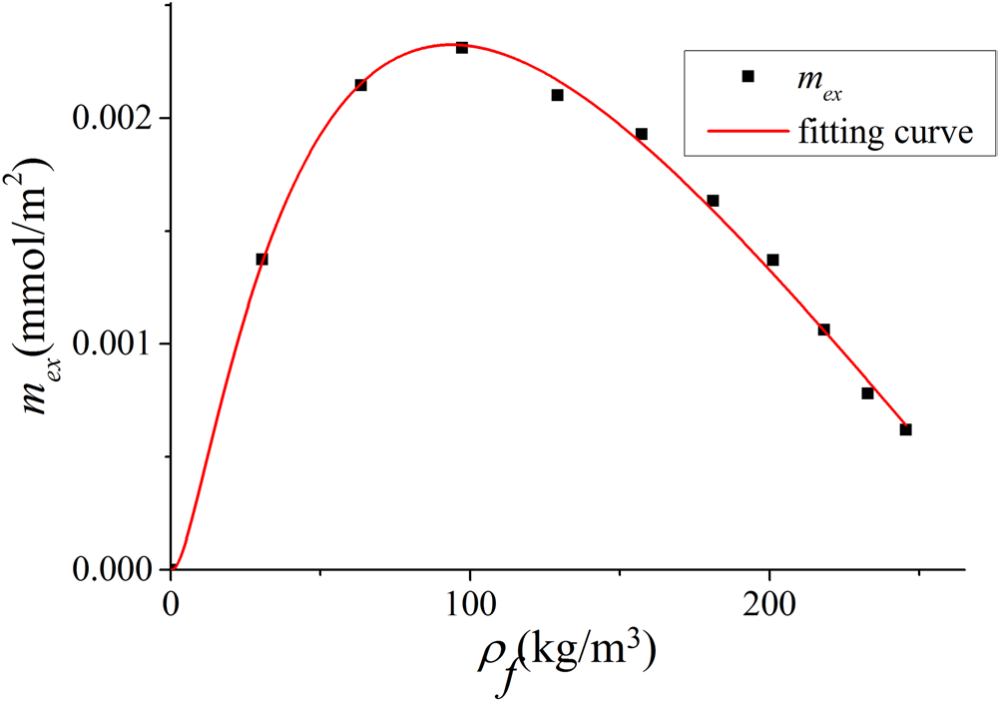
SDR Fitting with constant ${\rho }_{a}^{SDR{,}^{\ast \ast }}$ to the excess adsorption in illite nanopores of $W=4$ nm at 333.15 K with ${R}^{2}=0.9962$.

Our excess adsorption is fitted by the least-square method with all free parameters in Eq. (19) varying over the following ranges: $0 < {\rho }_{a}^{SDR{,}^{\ast }} < 500$ kg/m^3^, ${V}_{a,\,\max }^{SDR{,}^{\ast }} > 0$ nm^3^, and $0 < {C}^{\ast \ast } < 0.05$ mol^2^/kJ ^2^ ^31^. Excess adsorption in illite nanopores of $W=4$ nm at 333.15 K is used for SDR fitting with constant ${\rho }_{a}^{SDR{,}^{\ast \ast }}$ as shown in Fig. 18. The fitting parameters are ${\rho }_{a}^{SDR{,}^{\ast \ast }}=\mathrm{286}.09381$ kg/m^3^, ${V}_{a,\max }^{SDR{,}^{\ast }}=7.5316$ nm^3^, and ${C}^{\ast \ast }=0.02848$ mol^2^/kJ^2^, respectively. The fitted ${\rho }_{a}^{SDR{,}^{\ast \ast }}$ is less than the density of liquid methane at boiling temperature, 420 kg/m^3^ or the methane density at the critical point, 373 kg/m^3^, which were used by Xiong *et al*.^31^.

**Figure 19:**
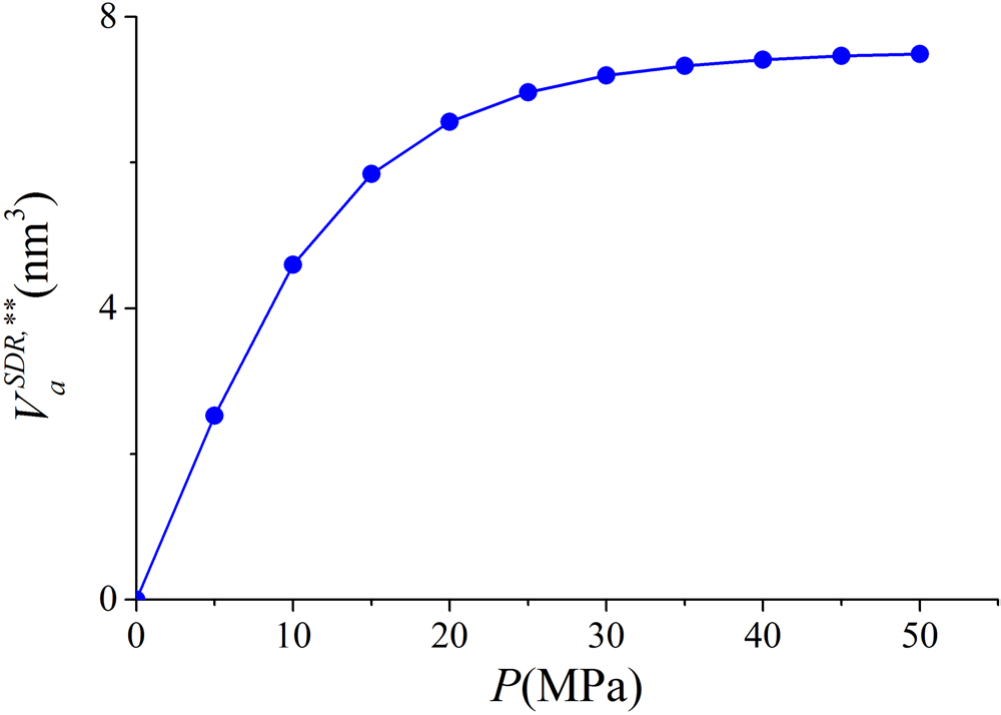
${V}_{a}^{SDR{,}^{\ast \ast }}$ for a given pressure from SDR model with constant ${\rho }_{a}^{SDR{,}^{\ast \ast }}$.

With constant ${\rho }_{a}^{SDR{,}^{\ast \ast }}$, the adsorbed phase volume ${V}_{a}^{SDR{,}^{\ast \ast }}$ for a given pressure from SDR fitting varies with pressure (bulk density) as in Eq. (20). We present ${V}_{a}^{SDR{,}^{\ast \ast }}$ for a given pressure in Fig. 19. It is observed that ${V}_{a}^{SDR{,}^{\ast \ast }}$ monotonically increases with pressure. At $P=5$ MPa, the calculated ${V}_{a}^{SDR{,}^{\ast \ast }}$ is 2.5224 nm^3^ and the width of adsorbed phase is only around 0.0852 nm. Such small adsorbed phase is unphysical, since at $P=5$ MPa, the width of adsorbed phase is much larger than that as shown in Fig. 8.

**Figure 20:**
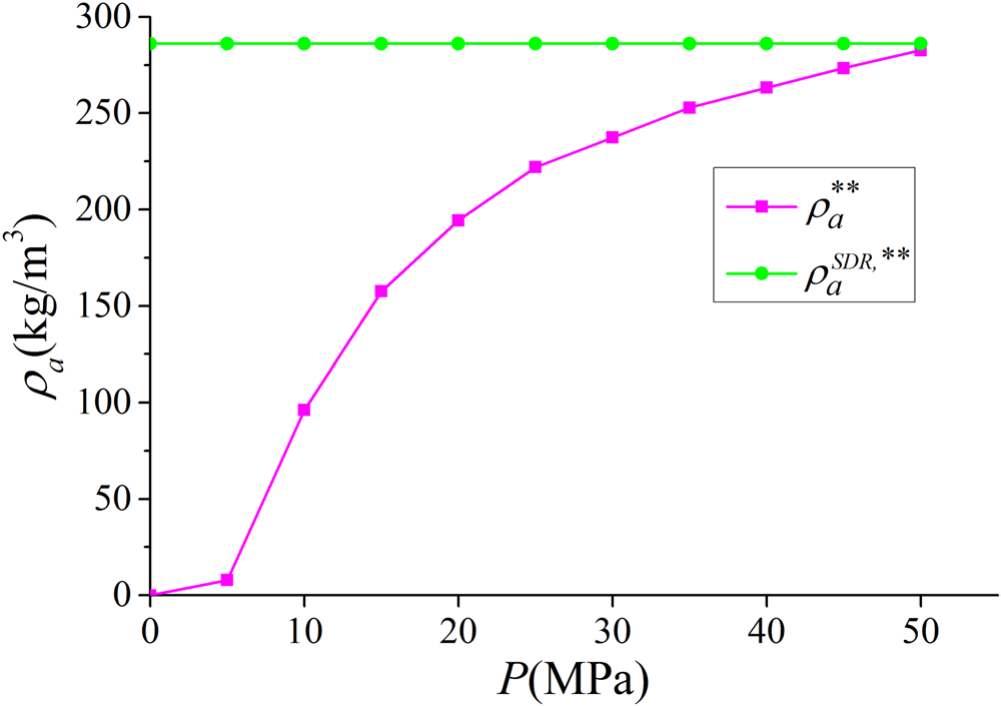
${\rho }_{a}^{\ast \ast }={\int }_{A}^{{B}^{\ast \ast }}\rho (z)dz/{z}_{A{B}^{\ast \ast }}$ and ${\rho }_{a}^{SDR{,}^{\ast \ast }}$ from SDR model with constant adsorbed phase density.

With the fitted ${V}_{a}^{SDR{,}^{\ast \ast }}$, we present the ${\rho }_{a}^{\ast \ast }={\int }_{A}^{{B}^{\ast \ast }}\rho (z)dz/{z}_{A{B}^{\ast \ast }}$ with varying adsorbed phase boundary ${B}^{\ast \ast }$ (${B}^{\ast \ast }$ is obtained from ${V}_{a}^{SDR{,}^{\ast \ast }}$ in Eq. (20)) and adsorbed phase width ${z}_{A{B}^{\ast \ast }}={V}_{a}^{SDR{,}^{\ast \ast }}/2{S}_{A}$ and ${\rho }_{a}^{SDR{,}^{\ast \ast }}$ in Fig. 20. Due to small ${V}_{a}^{SDR{,}^{\ast \ast }}$ at low pressure conditions, ${\rho }_{a}^{\ast \ast }$ is much smaller than the fitted value ${\rho }_{a}^{SDR{,}^{\ast \ast }}$. In addition, ${\rho }_{a}^{\ast \ast }$ varies with pressure, while ${\rho }_{a}^{SDR{,}^{\ast \ast }}$ is constant. In other words, ${\rho }_{a}^{SDR{,}^{\ast \ast }}$ from SDR model with constant adsorbed phase density is not self-consistent with methane density distributions.

**Figure 21:**
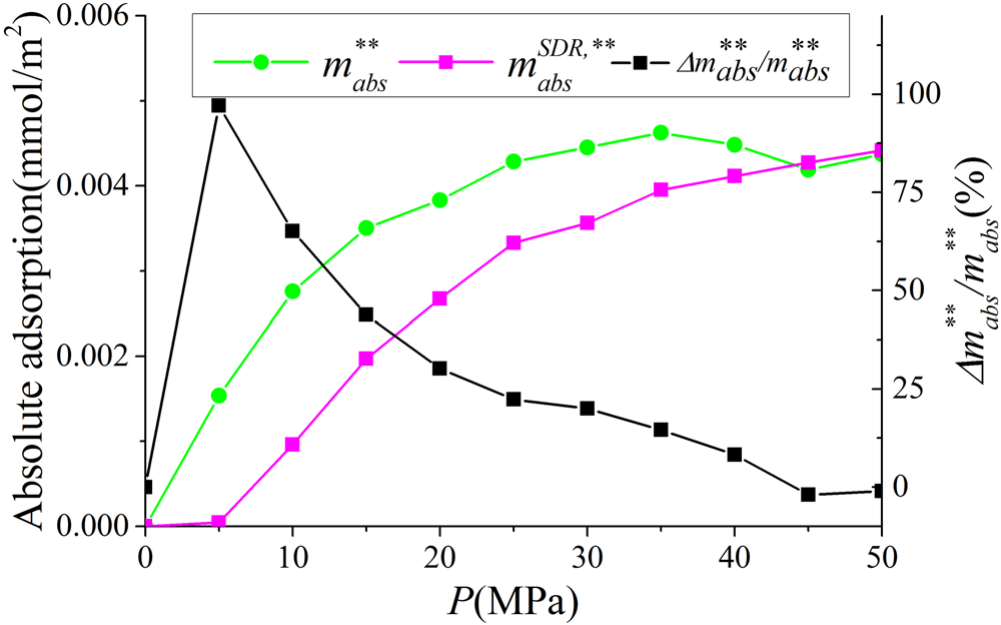
Absolute adsorption ${m}_{abs}^{\ast \ast }={\rho }_{a}^{\ast \ast }{V}_{a}^{SDR,\ast \ast }$ from the density distributions and ${m}_{abs}^{SDR,\ast \ast }={m}_{ex}/(1-{\rho }_{b}/{\rho }_{a}^{SDR,\ast \ast })$ from the SDR model with constant ${\rho }_{a}^{SDR,\ast \ast }$ as well as the variance of the absolute adsorption $\Delta {m}_{abs}^{\ast \ast }/{m}_{abs}^{\ast \ast }$.

We also present the absolute adsorption ${m}_{abs}^{\ast \ast }={\rho }_{a}^{\ast \ast }{V}_{a}^{SDR{,}^{\ast \ast }}$ from the density distributions and ${m}_{abs}^{SDR{,}^{\ast \ast }}={m}_{ex}/(1-{\rho }_{b}/{\rho }_{a}^{SDR{,}^{\ast \ast }})$ from the SDR model with constant ${\rho }_{a}^{SDR,\ast \ast }$ as well as the variance of the absolute adsorption ${\rm{\Delta }}{m}_{abs}^{\ast \ast }/{m}_{abs}^{\ast \ast }=({m}_{abs}^{SDR,\ast \ast }-{m}_{abs}^{\ast \ast })/{m}_{abs}^{\ast \ast }$ in Fig. 21. At low pressures, ${m}_{abs}^{\ast \ast }$ is much less than ${m}_{abs}^{SDR,\ast \ast }$ due to small adsorbed phase volume ${V}_{a}^{SDR,\ast \ast }$ and ${\rho }_{a}^{\ast \ast }$ as shown in Figs 19 and 20. The variance can be as large as 100% at 5 MPa. As pressure increases, the agreement becomes better.

#### Comparison between SDR Fittings with Our Model

In our model, we define the adsorbed phase width ${z}_{AB}$ as 0.38 nm which is close to the diameter of methane molecules. In SDR model with constant ${V}_{a}^{SDR,\ast }$, ${V}_{a}^{SDR,\ast }$ is obtained from the fitting with ${m}_{ex}$ and the resulting adsorbed phase width is ${z}_{A{B}^{\ast }}=0.29$ nm. In SDR model with constant ${\rho }_{a}^{SDR,\ast \ast }$, ${V}_{a}^{SDR,\ast \ast }$ varies with pressure, while the maximum adsorbed phase volume ${V}_{a,\,\max }^{SDR,\ast \ast }$ is obtained from the fitting with ${m}_{ex}$ and the resulting maximum adsorbed phase width is ${z}_{A{B}^{\ast \ast }}=0.25$ nm. The adsorbed phase volumes from both fitting approaches are less than methane diameter, which is considered as the width of adsorbed phase^68^.

**Figure 22:**
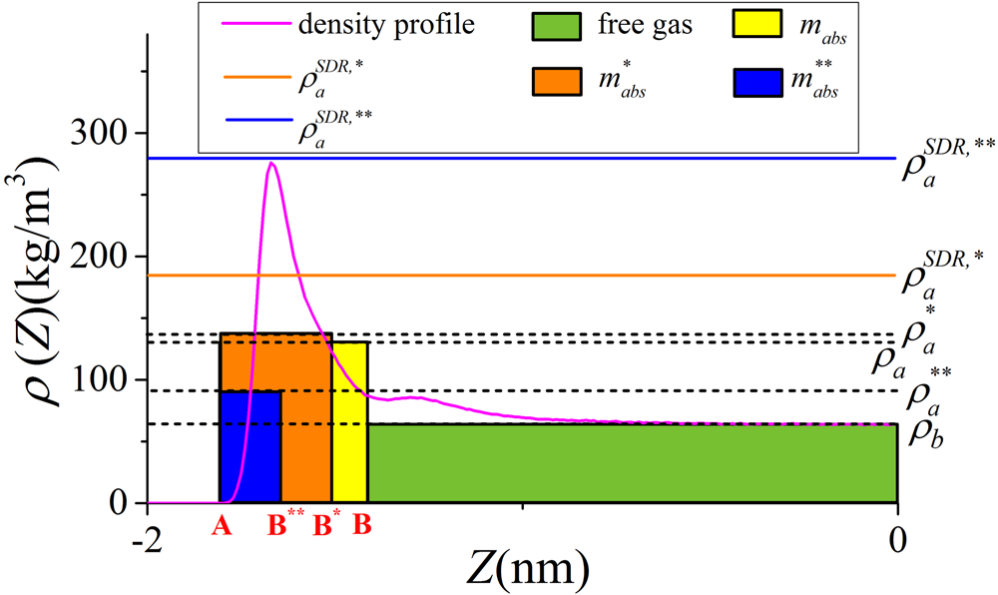
The schematic representation of adsorbed and free gas regions for methane adsorption in illite nanopore of $W=4$ nm at 333.15 K and 10 MPa. The yellow area presents the adsorbed phase from our model, the orange area presents the adsorbed phase from SDR model with constant ${V}_{a}^{SDR{,}^{\ast }}$, the blue area depicts the adsorbed phase from SDR model with constant ${\rho }_{a}^{SDR{,}^{\ast \ast }}$ with corresponding ${V}_{a}^{SDR{,}^{\ast \ast }}$ at 10 MPa, and the green area depicts the free gas phase from our model. B, B^*^, and B^**^ represent the boundaries of adsorbed phase from our model, SDR model with constant ${V}_{a}^{SDR,\ast }$, and SDR model with constant ${\rho }_{a}^{SDR{,}^{\ast \ast }}$, respectively. The heights of adsorbed and free gas phases from our model are depicted from ${\rho }_{a}={\int }_{A}^{B}\rho (z)dz/{z}_{AB}$ and ${\rho }_{b}$ from NIST Chemistry Webbook, respectively. The heights of adsorbed phase from SDR model with constant ${V}_{a}^{SDR{,}^{\ast }}$ and ${\rho }_{a}^{SDR{,}^{\ast \ast }}$ are depicted from ${\rho }_{a}^{\ast }={\int }_{A}^{{B}^{\ast }}\rho (z)dz/{z}_{A{B}^{\ast }}$ and ${\rho }_{a}^{\ast \ast }={\int }_{A}^{{B}^{\ast \ast }}\rho (z)dz/{z}_{A{B}^{\ast \ast }}$, respectively. The adsorbed phase density ${\rho }_{a}^{SDR{,}^{\ast }}$ from SDR model with constant ${V}_{a}^{SDR,\ast }$ is from Eq. (17), and ${\rho }_{a}^{SDR{,}^{\ast \ast }}=286.09381$ kg/m^3^ is from the SDR fitting with constant ${\rho }_{a}^{SDR{,}^{\ast \ast }}$.

In Fig. 22, we present the schematic representation of adsorbed phases from different approaches and the corresponding adsorbed phase density and volume of methane density distributions in illite nanopore of $W=4$ nm at 333.15 K and $P=10$ MPa. It can be seen that due to small ${V}_{a}^{SDR,\ast \ast }$ from SDR model with constant ${\rho }_{a}^{SDR,\ast \ast }$, the adsorbed phase from fitting can only cover part of adsorption layer. The actual adsorbed phase density ${\rho }_{a}^{\ast \ast }={\int }_{A}^{{B}^{\ast \ast }}\rho (z)dz/{z}_{A{B}^{\ast \ast }}$ is much smaller than the fitted value ${\rho }_{a}^{SDR,\ast \ast }$. As a result, there is large discrepancy between ${m}_{abs}^{\ast \ast }={\rho }_{a}^{\ast \ast }{V}_{a}^{SDR,\ast \ast }$ and ${m}_{abs}^{SDR,\ast \ast }={m}_{ex}/(1-{\rho }_{b}/{\rho }_{a}^{SDR,\ast \ast })$ as shown in Fig. 21. Comparing to ${V}_{a}^{SDR,\ast \ast }$ from SDR model with constant ${\rho }_{a}^{SDR,\ast \ast }$, ${V}_{a}^{SDR,\ast }$ from constant adsorbed phase volume fitting is larger and the calculated ${\rho }_{a}^{\ast }$ is closer to ${\rho }_{a}^{SDR,\ast }$. However, due to smaller adsorbed phase volume, comparing to our model, it has larger transition zone in free gas phase, which negatively affects the absolute adsorption calculation.

**Figure 23:**
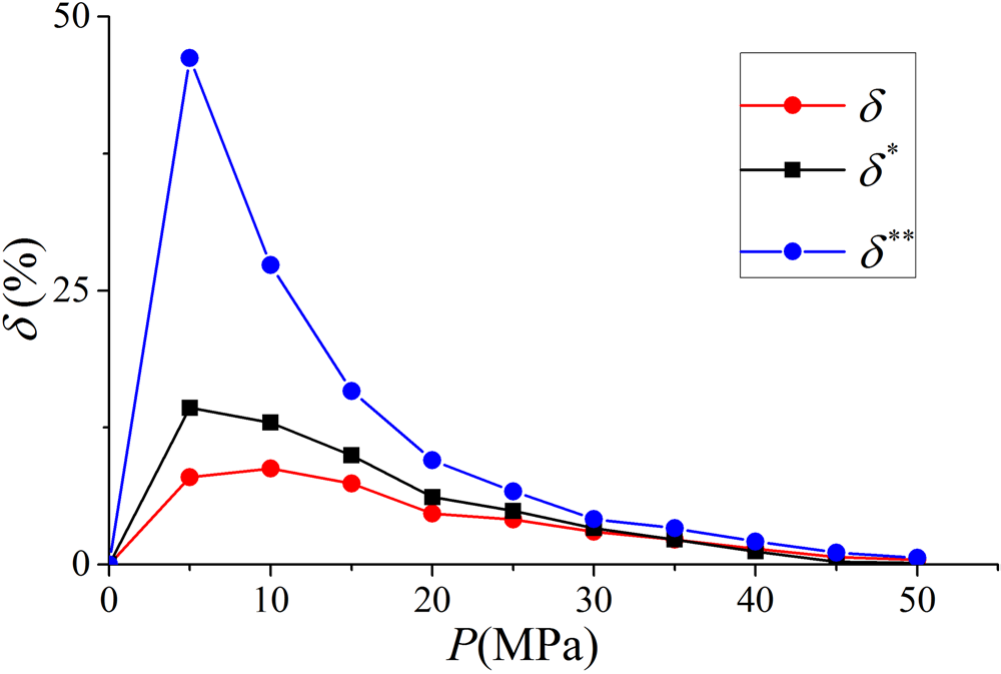
$\delta =({\int }_{B}^{B\text{'}}\rho (z)dz/{z}_{BB\text{'}}-{\rho }_{b})/{\rho }_{b}$ from our model, ${\delta }^{\ast }=({\int }_{{B}^{\ast }}^{B{^{\prime} }^{\ast }}\rho (z)dz/{z}_{{B}^{\ast}{B}^{\ast\prime}}-{\rho }_{b})/{\rho }_{b}$ from SDR fitting with constant ${V}_{a}^{SDR{,}^{\ast }}$, and ${\delta }^{\ast \ast }=({\int }_{{B}^{\ast \ast }}^{B{^{\prime} }^{\ast \ast }}\rho (z)dz/{z}_{{B}^{\ast \ast }{B}^{\ast \ast ^{\prime} }}-{\rho }_{b})/{\rho }_{b}$ from SDR fitting with constant ${\rho }_{a}^{SDR{,}^{\ast \ast }}$, respectively, in illite nanopore of $W=4$ nm at 333.15 K. ${z}_{BB\text{'}}$, ${z}_{B{\text{'}}^{\ast }B{\text{'}}^{\ast }}$, and ${z}_{B{\text{'}}^{\ast \ast }B{\text{'}}^{\ast \ast }}$ represent the width of free gas phase from our model, SDR model with constant ${V}_{a}^{SDR{,}^{\ast }}$, and SDR model with constant ${\rho }_{a}^{SDR{,}^{\ast \ast }}$, respectively.

Similar to Fig. 7, in Fig. 23, we present the variance of the average density in the free gas phase from ${\rho }_{b}$ in our model and SDR fittings. It is observed that due to small ${V}_{a}^{SDR{,}^{\ast \ast }}$, SDR model with constant ${\rho }_{a}^{SDR{,}^{\ast \ast }}$ has the largest variance. The variance can be as much as 50% at low pressure conditions. Comparing to SDR model with constant ${V}_{a}^{SDR{,}^{\ast }}$, the variance in our model is smaller. In other words, the effect of transition zone is less significant in our model.

Overall, SDR model with constant adsorbed phase volume performs better than that with constant adsorbed phase density. The latter may not have solid physical foundation. The adsorbed phase densities from SDR method are inconsistent with density distributions. Comparing to both fitting methods, our model agrees better with the adsorption model shown in Fig. 1.

### Comparison of Excess and Absolute Adsorption in Various Clay Minerals

In this subsection, we will compare the excess and absolute adsorption in various clay nanopores.

**Figure 24:**
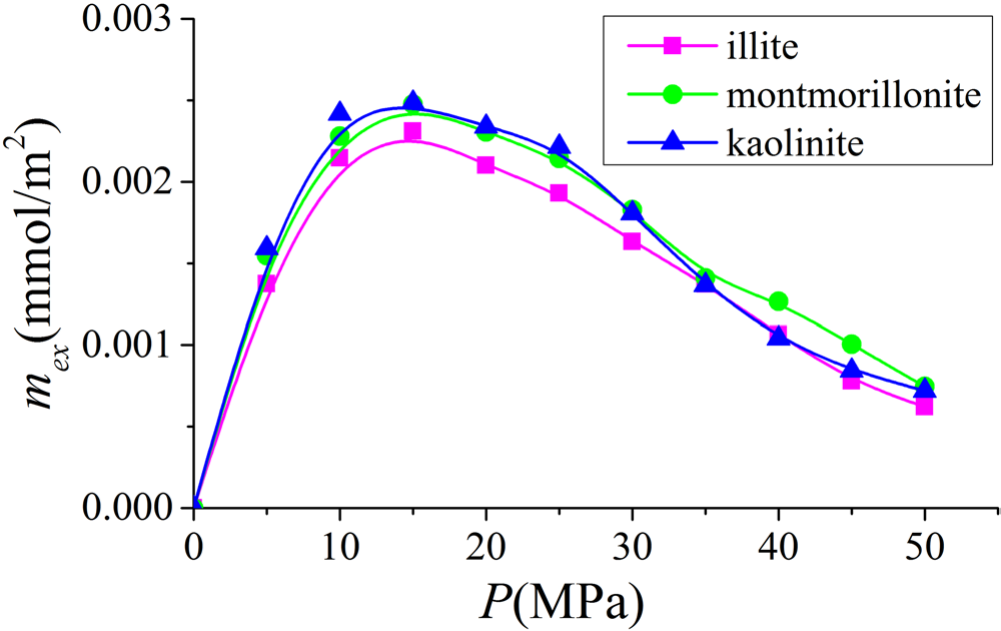
Excess adsorption in illite, montmorillonite and kaolinite nanopores of $W=4$ nm at 333.15 K.

**Figure 25:**
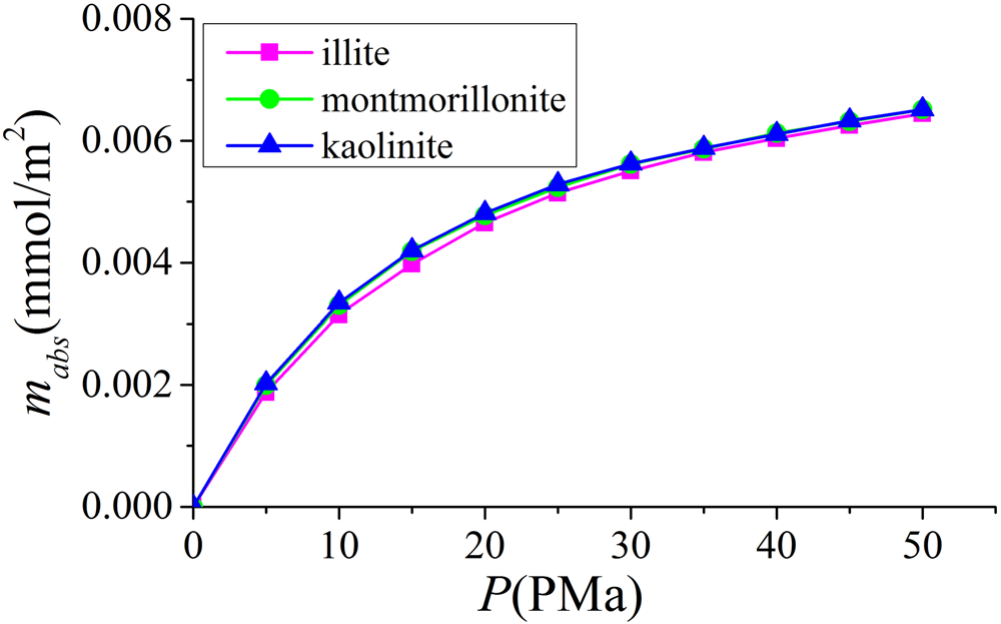
Absolute adsorption in illite, montmorillonite and kaolinite nanopores of $W=4$ nm at 333.15 K.

In Fig. 24, we present the methane excess adsorption in illite, montmorillonite, and kaolinite nanopores at 333.15 K. As shown in Fig. 3, when $W\ge 2$ nm, ${m}_{ex}$ per SSA becomes insensitive to the pore size. Thus, we only present the results for 4 nm pore. We observe that ${m}_{ex}$ per SSA is similar for different clay minerals at given pressures. By using volumetric method, Ji *et al*.^14^ found that methane excess adsorption for various types of clay minerals correlates well with SSA. For various clay minerals, excess adsorption capacity has a maximum around 15 MPa. As pressure further increases, excess adsorption decreases due to higher ${\rho }_{b}$. In Fig. 25, we present the corresponding absolute adsorption in various clay minerals. The adsorbed phase region in montmorillonite and kaolinite nanopores is defined in the same way as illite at 50 MPa as shown in Fig. 6. The absolute adsorption is obtained from ${m}_{ex}$ and ${V}_{a}$ as in Eq. (3). Similar to the excess adsorption, the absolute adsorptions per SSA in various clay nanopores are similar.

### Effect of Temperatures on Excess and Absolute Adsorption

**Figure 26:**
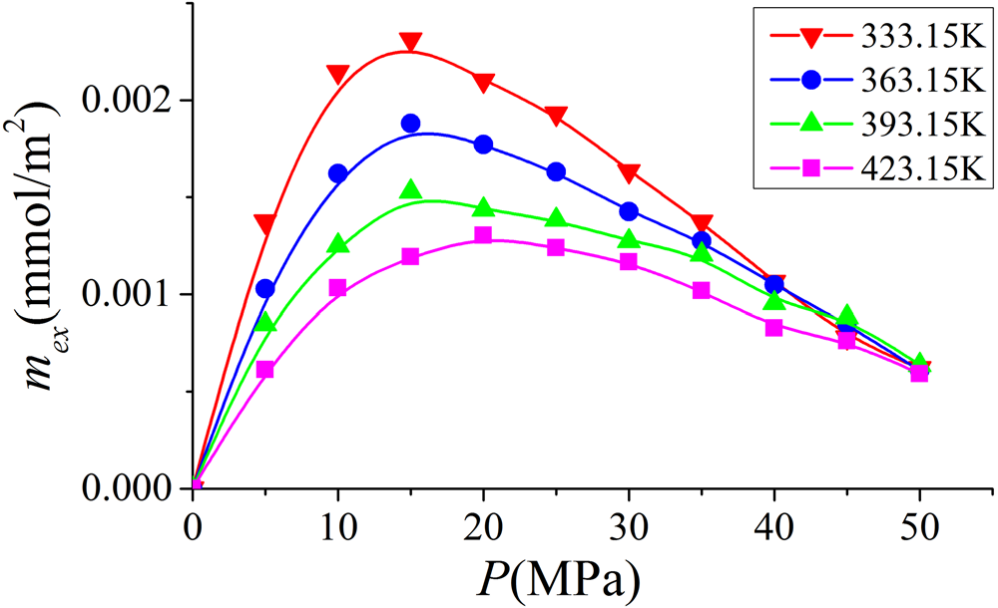
Excess adsorption in illite nanopores of $W=4$ nm at various temperatures.

**Figure 27:**
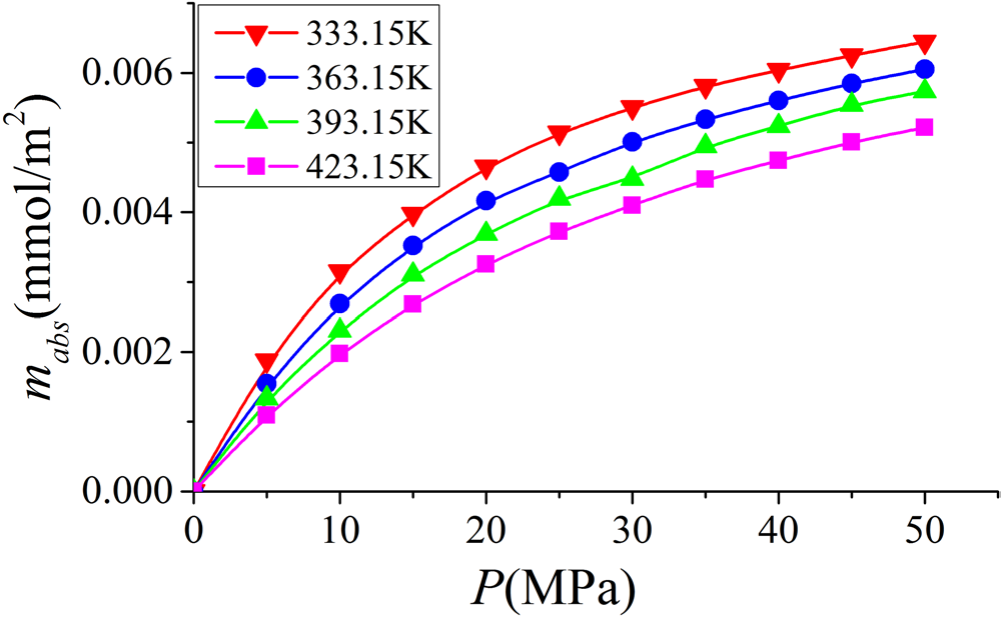
The same as Fig. 26, but for absolute adsorption.

In Fig. 26, we present the excess adsorption isotherms at various temperatures in illite nanopores of 4 nm pores. We observe that in the low pressure region, as temperature increases, the excess adsorption amount decreases. But in high pressure region, the excess adsorption becomes comparable for various temperatures. However, the absolute adsorption decreases with temperature as shown in Fig. 27. It is because at higher temperature, due to weaker fluid-surface interactions, surface adsorption becomes less significant.

## Conclusion

In this work, we use GCMC simulations to study methane adsorption in illite, montmorillonite and kaolinite nanopores of varying pore sizes and temperatures. Clay minerals can have micro-to-mesopore structures which can greatly enhance gas-in-place in shale reservoirs. We employ full atomistic models to describe clay nanopore structures and explicitly consider the intermolecular interactions. To match with experimental data^14^, we use helium adsorption to determine the effective pore volume and the corresponding excess adsorption.

Our simulation shows a good agreement with experimental data on the methane excess adsorption. When $W\ge 2$ nm, the excess adsorption per SSA becomes insensitive to the pore size. We find that SSA plays a dominant role in the methane excess and absolute adsorption capacity in various clay minerals in line with previous experimental data^14^. The methane density distributions in various clay minerals indicate that adsorbed phase density is not only dependent on temperature but also pressure. As a result, using a constant liquid phase density to calculate absolute adsorption may become inapplicable. In addition, we find that the absolute adsorption obtained from the excess adsorption and adsorbed phase density may bring a significant error. Instead, we propose to use the excess adsorption and adsorbed phase volume. While experiments may only get total or excess adsorption, molecular simulation can provide ${V}_{a}$ of the specific adsorbate based on the density distribution.

We also show that the SDR method used in experiment to correct the excess adsorption to the absolute adsorption may result in unphysical values for adsorbed phase density or volume, especially for SDR model with constant adsorbed phase density assumption.

This work should shed important insights into the evaluation of absolute adsorption and provide fundamental understandings toward the underlying mechanisms of methane adsorption in clay nanopores.

## Electronic supplementary material

Supplementary Information
