# Supplementary material for: Characterization of Methane Excess and Absolute Adsorption in Various Clay Nanopores from Molecular Simulation
Source: Sci Rep. 2017 Sep 20;7:12040. doi: 10.1038/s41598-017-12123-x (PMC5607345; doi:10.1038/s41598-017-12123-x)
Supplement: Supplementary file 1 — Supplementary Information [file 41598_2017_12123_MOESM1_ESM.pdf]

Supplementary Information

Characterization of Methane Excess and Absolute Adsorption in Various Clay Nanopores from Molecular Simulation

Yuanyuan Tian[1,2,3], Changhui Yan[1,2], and Zhehui Jin[3]*

[1]State Key Laboratory for Oil and Gas Reservoir Geology and Exploitation (Chengdu University of Technology), Chengdu, 610059, Sichuan, P.R. China

[2]College of Energy, Chengdu University of Technology, Chengdu, 610059, Sichuan, P.R. China

[3]School of Mining and Petroleum Engineering, Faculty of Engineering, University of Alberta, Edmonton, T6G 1H9, Canada

A. Helium Adsorption in Clay Minerals to Determine the Effective Pore Volume

In Figure SI.A1, we present the density distributions of helium molecules in 4 nm illite nanopores at 333.15 K with varying bulk pressures. We observe that the helium adsorption layer is negligible. Near the pore surface, density approaches zero, meaning that V_p is smaller than $W \times S_A$. It is because the helium molecule has its own size that the center of mass cannot lay on the surface. We also present the $\langle N_{He} \rangle$ versus $\rho^m_{He,b}$ in Figure SI.A2. It can be seen that for various clay minerals, $\langle N_{He} \rangle$ and $\rho^m_{He,b}$ show excellent linear correlation with $R^2 > 0.9999$. The excellent linear correlation indicates the effective pore volume is independent of pressure. We also present the $\langle N_{He} \rangle$ versus $\rho^m_{He,b}$ for 4 nm illite nanopores at different temperature in Figure SI.A3, respectively. The perfectly overlapped lines show that effective pore volume is independent of temperature. We present the calculated V_P versus P at various temperatures in Figure SI.A4. It is observed that V_P is independent of pressure and temperature.

* Corresponding Author: zhehui2@ualberta.ca

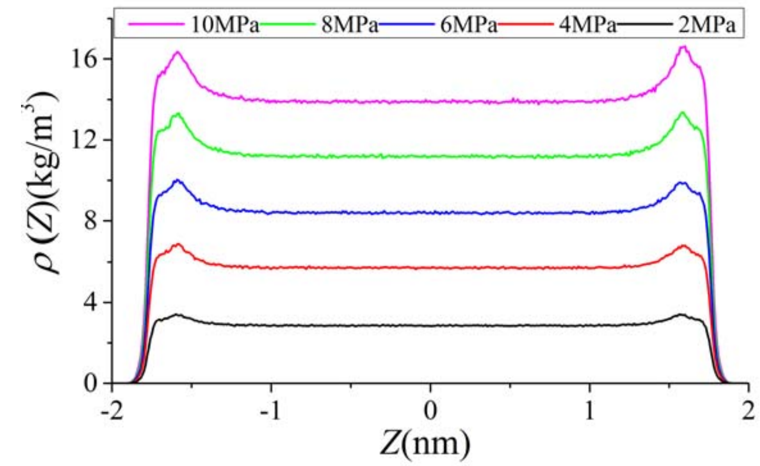

Figure SI.A1 Helium density distributions in illite nanopores of $W = 4$ nm and $T = 333.15$ K

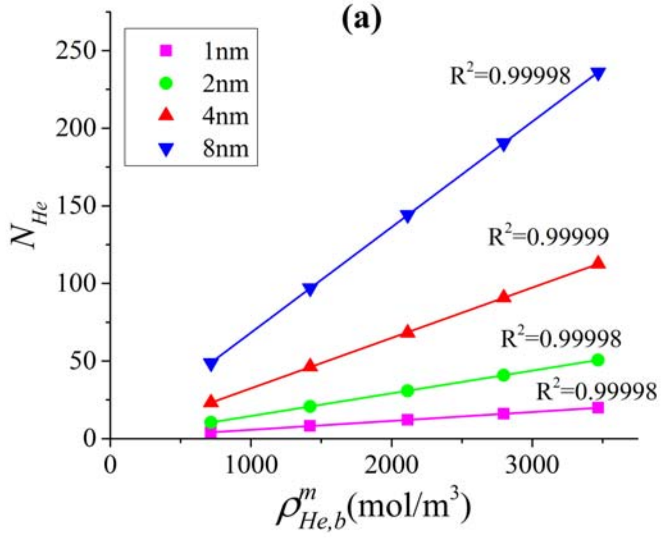

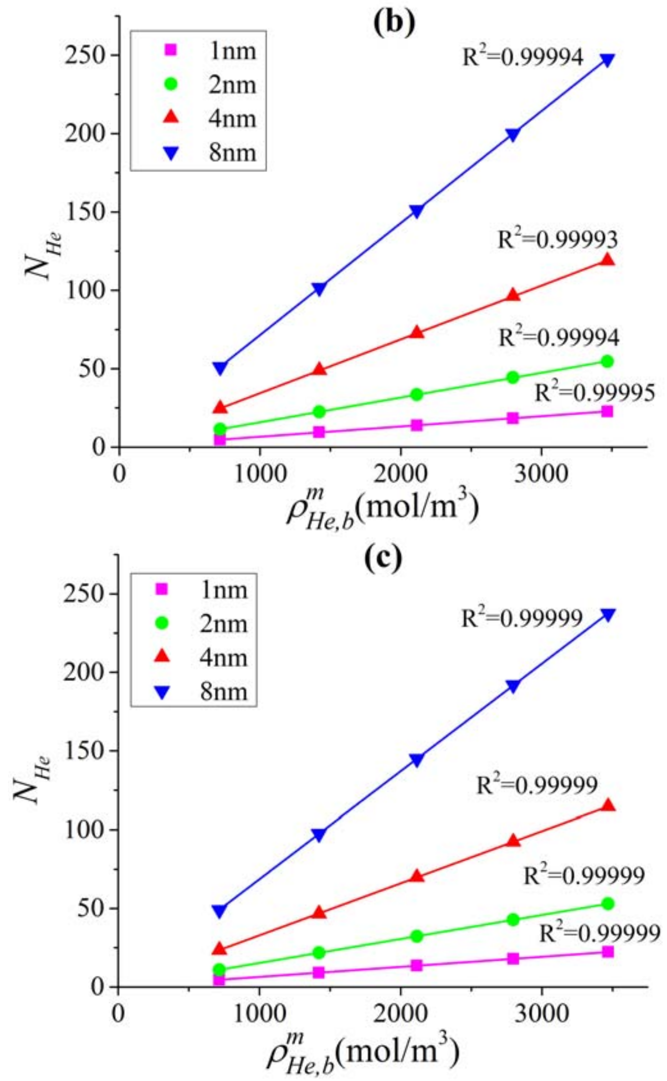

Figure SI.A2 Total helium uptake versus bulk density in (a) illite; (b) montmorillonite; (c) kaolinite nanopores at 333.15 K. The solid lines are guidance lines.

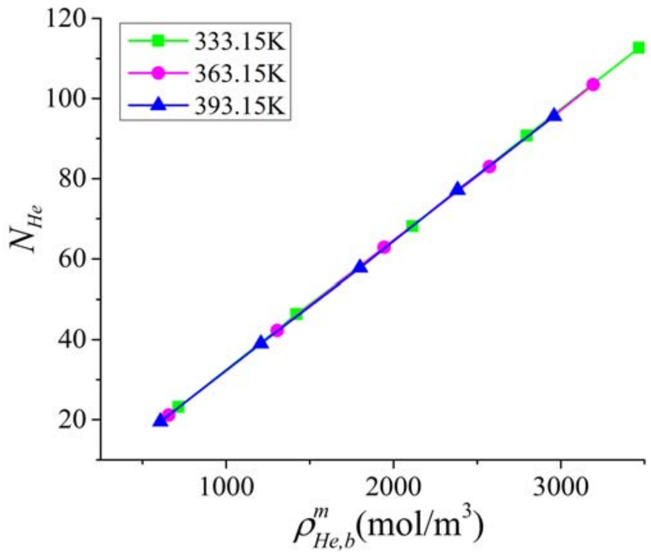

Figure SI.A3 Total helium uptake versus bulk density in 4 nm illite at 333.15K, 363.15K and 393.15K. The solid lines are guidance lines.

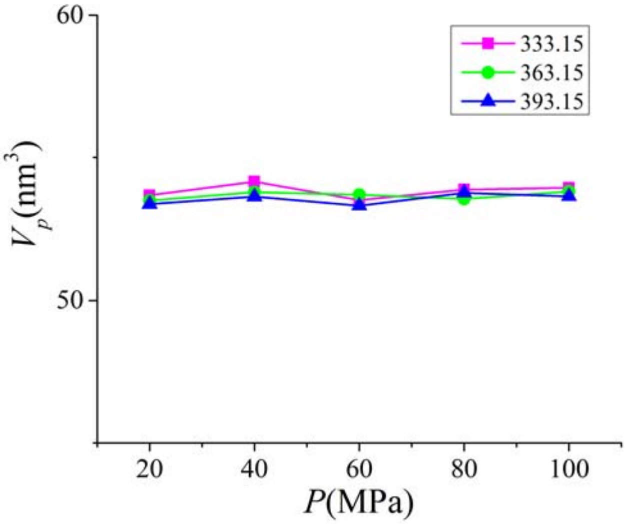

Figure SI.A4 Effective pore volume versus pressure in 4 nm illite at 333.15K, 363.15K and 393.15K.

B. Absolute Adsorption at High Pressures

In Figure SI.B1, we present $m_{abs} = \rho_a V_a$ and $m_{abs,1} = m_{ex} + \rho_b V_a$ up to 80 MPa. We observe that m_{abs} continuously increases with pressure even up to 80 MPa. It further confirms that determining adsorbed phase volume by slope of absolute adsorption isotherm is not

applicable. Furthermore, the agreement between m_{abs} and $m_{abs,1}$ is better at higher pressures and the variance $\Delta m_1 / m_{abs}$ is only around 2%. The excellent agreement is due to negligible difference between ρ_f^a and ρ_b as shown in Figure SI.B2. At high pressures, the variance δ is less than 0.5%.

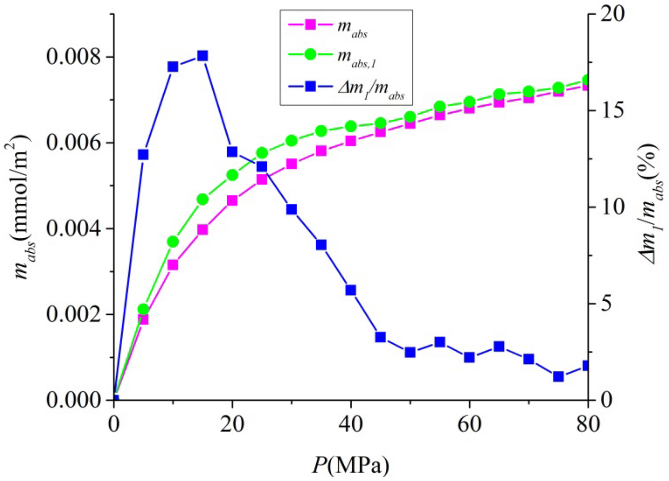

Figure SI.B1 Absolute adsorption isotherms in illite nanopores of $W = 4$ nm at 333.15K with pressure 0~80 MPa and difference between $m_{abs,1}$ and m_{abs}. $m_{abs} = \rho_a V_a$ is based on density distribution and $m_{abs,1} = m_{ex} + \rho_b V_a$ is from Eq. (3).

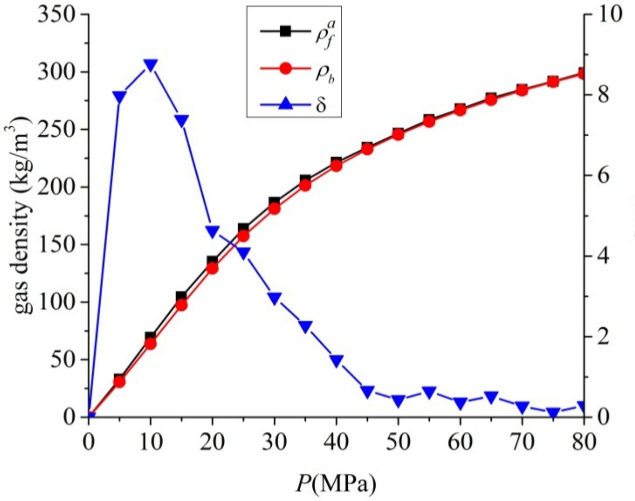

Figure SI.B2 Comparison of ρ_f^a and ρ_b from NIST and δ for methane adsorption in illite nanopore of $W = 4$ nm at 333.15 K and pressure up to 80 MPa.
